# OptZyme: Computational Enzyme Redesign Using Transition State Analogues

**DOI:** 10.1371/journal.pone.0075358

**Published:** 2013-10-07

**Authors:** Matthew J. Grisewood, Nathanael P. Gifford, Robert J. Pantazes, Ye Li, Patrick C. Cirino, Michael J. Janik, Costas D. Maranas

**Affiliations:** 1 Department of Chemical Engineering, The Pennsylvania State University, University Park, Pennsylvania, United States of America; 2 Department of Chemical and Biomolecular Engineering, The University of Houston, Houston, Texas, United States of America; Oak Ridge National Laboratory, United States of America

## Abstract

OptZyme is a new computational procedure for designing improved enzymatic activity (i.e., k_cat_ or k_cat_/K_M_) with a novel substrate. The key concept is to use transition state analogue compounds, which are known for many reactions, as proxies for the typically unknown transition state structures. Mutations that minimize the interaction energy of the enzyme with its transition state analogue, rather than with its substrate, are identified that lower the transition state formation energy barrier. Using *Escherichia coli* β-glucuronidase as a benchmark system, we confirm that K_M_ correlates (R^2^ = 0.960) with the computed interaction energy between the enzyme and the *para*-nitrophenyl- β, D-glucuronide substrate, k_cat_/K_M_ correlates (R^2^ = 0.864) with the interaction energy of the transition state analogue, 1,5-glucarolactone, and k_cat_ correlates (R^2^ = 0.854) with a weighted combination of interaction energies with the substrate and transition state analogue. OptZyme is subsequently used to identify mutants with improved K_M_, k_cat_, and k_cat_/K_M_ for a new substrate, *para*-nitrophenyl- β, D-galactoside. Differences between the three libraries reveal structural differences that underpin improving K_M_, k_cat_, or k_cat_/K_M._ Mutants predicted to enhance the activity for *para*-nitrophenyl- β, D-galactoside directly or indirectly create hydrogen bonds with the altered sugar ring conformation or its substituents, namely *H162S*, *L361G*, *W549R*, and *N550S*.

## Introduction

Enzymes are highly-specific, biomolecular catalysts that cause extraordinary reaction rate enhancements under mild conditions [1]. Enzyme activity is of paramount importance in the economics of cellulosic ethanol (and other biofuels) production [2], [3]. Improving enzymatic activity is generally carried out using primarily experimental techniques (i.e., directed evolution strategies) relying on screening large combinatorial libraries [4]. Experiments can be synergistically coupled with efficient computational screening protocols (i.e., fine-tuning of *in silico* mutants with random mutagenesis) to identify mutants within promising regions of the sequence space for constructing enriched libraries. Reliable computational techniques for identifying mutations that lead to enzymatic activity improvements would have a cross-cutting impact on many fronts ranging from biofuel production and biomass pretreatment to pro-drug activation and the design of new therapeutics [5]–[8].

Various computational tools utilizing primary, secondary, and/or tertiary protein structural information have been tested to discover promising enzyme redesigns. These approaches range from relatively simple (e.g., comparative modeling [9]–[12] and scoring-based methods [13]–[19]) to complex (e.g., molecular mechanics force fields [20]–[26] and hybridized quantum mechanics/molecular mechanics (QM/MM) techniques [1], [27]–[33]). As the degree of complexity increases, there are often accuracy improvements at the expense of greater computational time. Even with all of these available methods, the computational design of enzymes remains a formidable task with only isolated successes [1], [23], [25], [26], [28]–[35] verified by experiment. A number of review articles [36], [37] highlight recent progress and remaining challenges in computational enzyme design.

Here, we introduce a new enzyme design method, OptZyme, to address some of these challenges. OptZyme uses transition state analogues (TSAs) as proxies for the typically unknown rate-limiting transition state (TS) structures. TSAs are potent inhibitors with a stable enzyme-bound complex that closely resemble the TS of an enzymatic reaction [38], [39]. TSAs manage to interfere with the enzyme catalytic activity by mimicking the geometry of the TS and preferentially binding with the enzyme over the substrate, thus preventing the reaction from proceeding. TSAs are known for many enzymatic reactions [40]–[43]. Improving catalysis by lowering the TS energy barrier can theoretically be achieved by identifying mutations that minimize the binding energy (BE) of the enzyme with its TSA, rather than with its substrate. We approximate BE with interaction energy (IE) to limit the force-field’s role in reconfiguring the free enzyme/substrate. The developed theoretical framework assumes that solute entropic changes and conformational changes upon binding are relatively small and that product release after the rate-limiting step is energetically favored. The concept of using TSAs for enzyme redesign has been previously explored [23], [44]. However, OptZyme is unique as it provides a theoretical framework for making use of TSA calculations to inform enzyme design while also integrating preliminary quantum mechanics (QM) information (e.g., rate-limiting step identification and ligand partial charge information).

Enzyme optimization using OptZyme can be achieved by designing libraries of mutations that raise k_cat_ or lower K_M_ within the Michaelis-Menten kinetic representation. K_M_ is related to the IE with the substrate, while k_cat_/K_M_ is expressed as a function of the IE with the TSA. We used OptZyme to redesign *Escherichia coli* β-glucuronidase (GUS) to favor the new substrate, *para*-nitrophenyl-β, D-galactoside (pNP-GAL) in place of *para*-nitrophenyl- β, D-glucuronide (pNP-GLU). pNP-GLU was used as a proxy for the native substrate (i.e., glycosaminoglycans containing glucuronic acid [45], [46]). Separate computational library designs were identified that optimize K_M_, k_cat_, or k_cat_/K_M_, and the observed differences were analyzed. Mutations *H162S*, *D163K*, *L361R*, *L361E*, *W549R,* and *N550S* were identified that optimized at the same time K_M_, k_cat_, and k_cat_/K_M_ for pNP-GAL instead of pNP-GLU. Mutations that (either directly or indirectly) created hydrogen bonds with the altered geometry of the TSA of the new substrate accounted for the majority of redesigns.

## Methods

### Redesign of GUS

**Figure 1:**
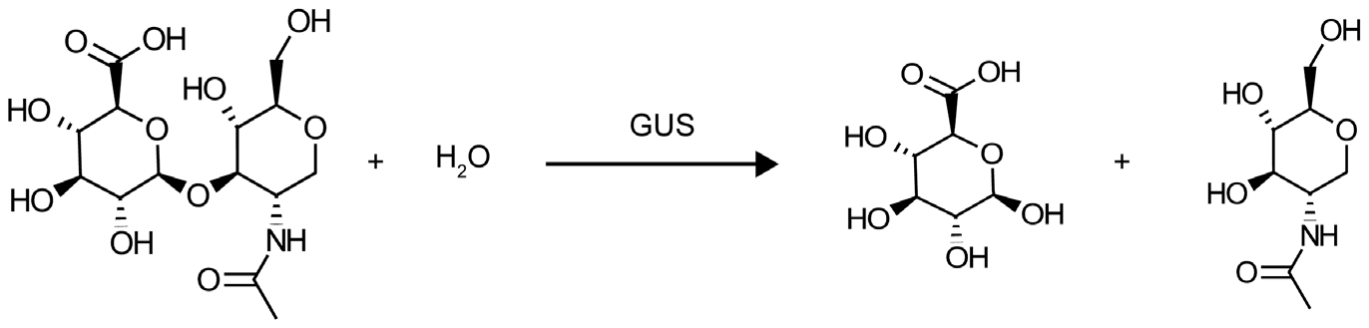
Native Reaction for GUS. GUS catalyzes the hydrolysis of a glucuronic acid-containing glycosaminoglycan to form two products, glucuronic acid and an amino sugar (acetylglucosamine in this reaction). pNP-GLU is used as the substrate instead of a glycosaminoglycan because *para*-nitrophenolate absorbance allows for spectroscopic monitoring of activity in experimental studies. Experimental activity measurements for GUS variants are used for verifying correlations between activity and IE.

**Figure 2:**
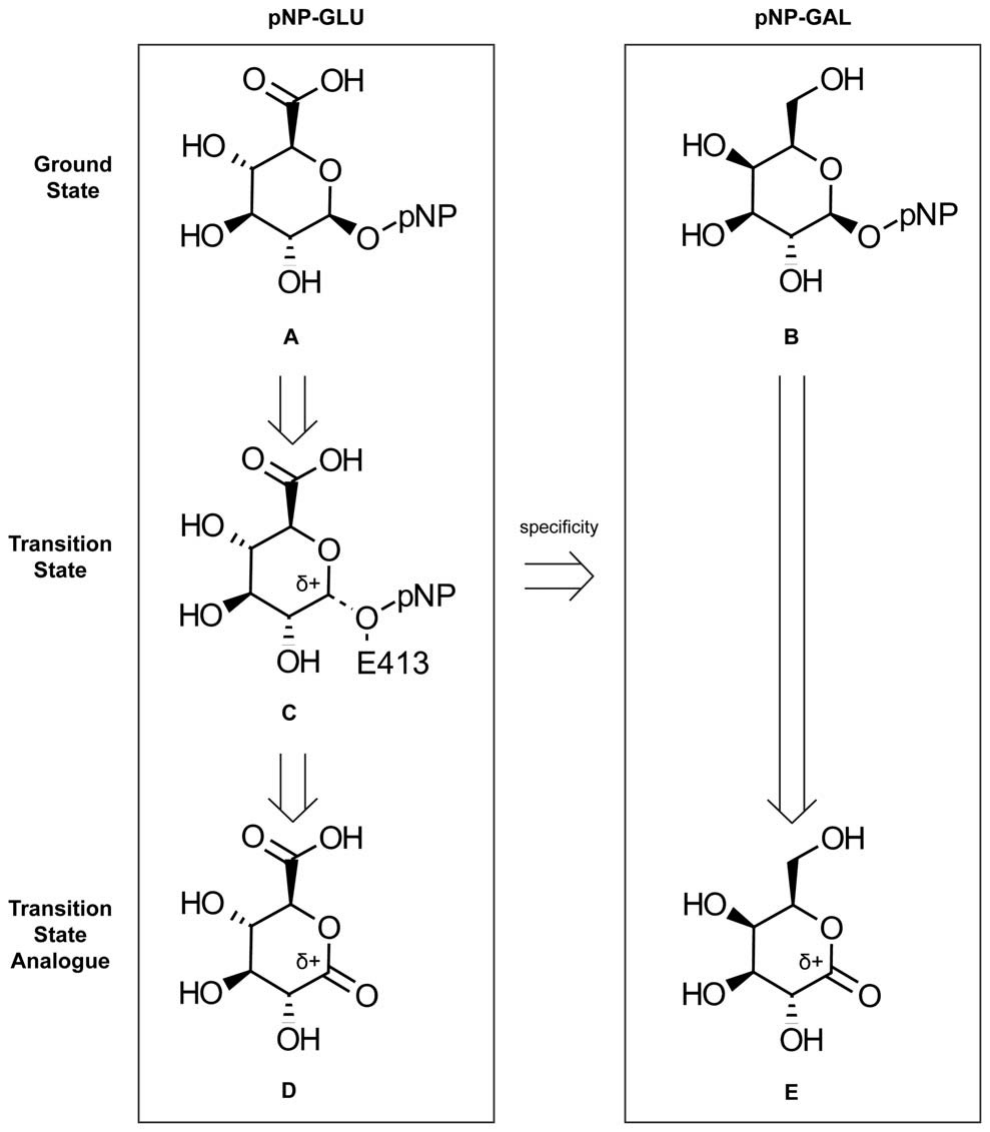
Comparison between ground state, hypothetical TS, and TSA for pNP-GLU and pNP-GAL. Differences between pNP-GLU (*A*) and pNP-GAL (*B*) include reversal of the stereospecificity of the C4 carbon and replacement of a carboxylic acid (pNP-GLU) at the C5 carbon with an alcohol (pNP-GAL). The previously-suggested [52], [53] TSA for pNP-GLU, 1,5-glucarolactone (*D*), resembles the proposed TS (*C*) in terms of charge distribution and stereospecificity of the carbohydrate. In contrast to the proposed TS structure, the TSA lacks the *para*-nitrophenyl (pNP) moiety and a hydrogen atom from the C1 carbon. In addition, the TSA (*D*) differs from pNP-GLU (*A*) by assuming a more flattened sugar ring geometry (see Figure S1 for dihedral angles) and partial positive charge at the anomeric carbon. The TSA for pNP-GAL, 1,5-galactonolactone (*E*), is similar to 1,5-glucaronolactone (*D*). The differences between 1,5-galactonolactone and 1,5-glucaronolactone are identical to the differences between pNP-GAL and pNP-GLU.

The design concept explored by OptZyme is to attempt to lower the TS barrier by optimally redesigning the enzyme so as to improve the binding affinity (approximated using IE) of a TSA. The native reaction for GUS is hydrolysis of glucuronic acid from the non-reducing end of the glycosaminoglycan [45], [46] (Figure 1). The native substrate more closely resembles pNP-GLU than pNP-GAL as seen in the structures of their sugar moieties (see Figure 2). pNP-GLU was used here in lieu of the native substrate as in previous experimental work [47]–[49] because its *para*-nitrophenolate leaving group is facile to spectrophotometric monitoring.

**Figure 3:**
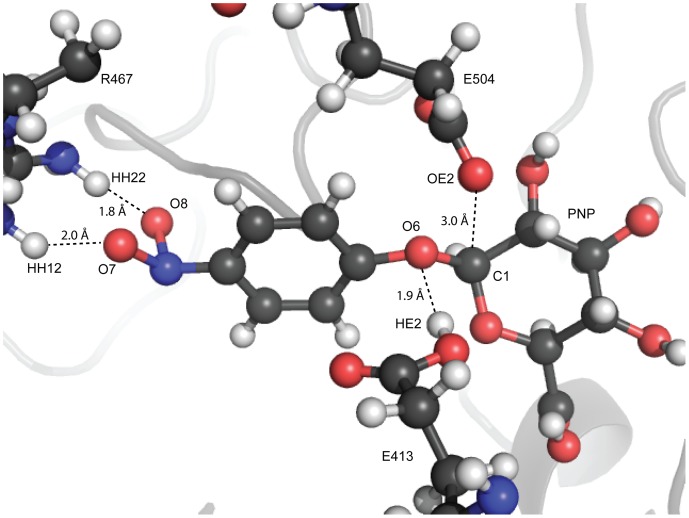
Active site geometry and restrained catalytic contacts. The active site is depicted in a ball-and-stick representation (C = black, O = red, N = blue, H = white). The nonbonded interactions seen reflect the distances restrained (as listed in Table 1). Key catalytic residues are labeled by their one-letter amino acid abbreviation followed by their position number. *para*-nitrophenyl- β, D-glucuronide (pNP-GLU) is labeled by the abbreviation “PNP” (see Figure 1). Atoms involved in restraints are labeled, along with interatomic distances.

The structure for GUS was computationally assembled largely from its unbound crystal structure (PDB: 3K46 [50]). A six-residue loop was not spatially resolved in PDB 3K46. The loop had to be modeled due to its proximity to the active site (minimum loop-substrate interatomic distance = 7.5 Å) and interactions with the substrate. An inhibitor-bound structure (PDB: 3LPF [50]) was used to obtain a reasonable conformation of the six-residue loop and pinpoint the binding site for pNP-GLU. The CHARMM [20] force field was used during energy minimizations while Nuclear Overhauser Effect (NOE) restraints were imposed between important catalytic residues (Table 1, Figure 3). The restraints were used to ensure conservation of the optimal catalytic geometry [51].

**Table 1:** NOE restraints applied during CHARMM energy minimization.

Atom 1	Atom 2	Minimum (Å)	Maximum (Å)	k_min_	k_max_
GLU 413: HE2	PNP: O6	1.7	1.8	75.0	100.0
GLU 504: OE2	PNP: C1	2.5	2.6	75.0	100.0
ARG 467: HH12	PNP: O7	1.7	1.8	75.0	100.0
ARG 467: HH22	PNP:O8	1.7	1.8	75.0	100.0

Restraints were placed on key catalytic contacts, determined from previous experimental [51] and preliminary QM information. Distances between atoms were selected based on typical nonbonded interaction lengths, and spring constants were determined iteratively so that the distances were properly restrained while not over-constraining the system. k_min_ was the harmonic constant implemented if the interatomic distance was too small, and k_max_ was the harmonic constant used if the interatomic distance was too large. k_min_<k_max_ because catalytic contacts would remain intact at smaller distances. Entries are shown in Figure 3.

**Figure 4:**
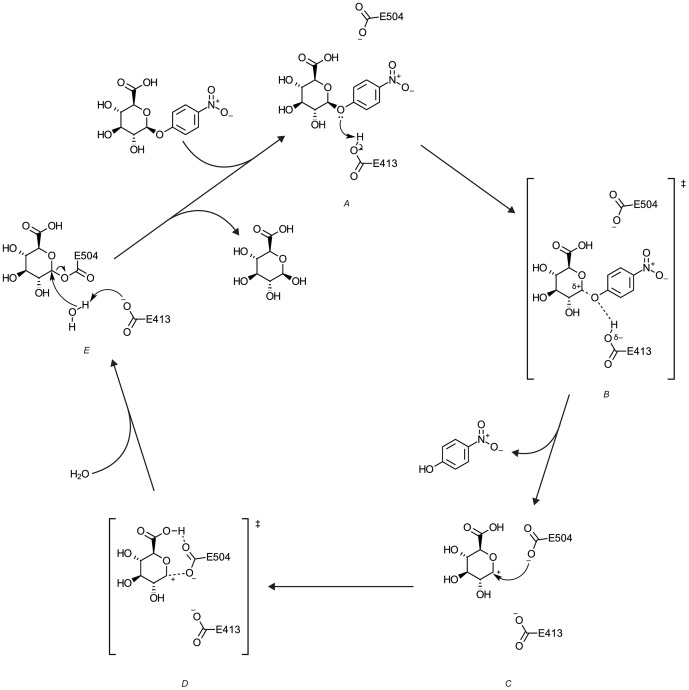
Proposed catalytic reaction mechanism of GUS from *in vacuo* QM calculations (Text S1). In the first step, substrate binds to the active site of GUS. Next, the lone pair on the glycosidic bond attacks the proton of E413 (*A*). This forms a hypothetical TS (*B*) with the glycosidic bond partially broken. The glycosidic bond is fully cleaved, releasing *para*-nitrophenolate and forming a carbocation intermediate (*C*). The electrons on the anionic E504 then attack the anomeric carbon, resulting in a hypothetical TS (*D*) where the carbocation and E504 are electrostatically attractive. A covalent intermediate (*E*) is formed between the carbohydrate moiety of pNP-GLU and E504. Presumably, in the next step, the basic E413 attacks a proton of a water molecule. The resulting hydroxide anion attacks the anomeric carbon to yield the product of the reaction. The two catalytic residues are regenerated for further turnover.

Upon modeling the GUS structure, the next step involved identifying a TSA. To our knowledge, the TS structure for the glycosidic hydrolysis of pNP-GLU is unknown, but there is information available on TSAs for GUS (i.e., 1,5-glucarolactone) [52], [53]. QM calculations were used to explore the reaction mechanism (see Figure 4) to aid in identifying the rate-limiting TS. We hypothesized a TS that has sp^2^ hybridization at the anomeric carbon because QM calculations confirmed the carbenium nature of the intermediate. Vibrational confirmation of the equilibrium states was not performed as structural constraints placed on the GUS residues prevents vibrational confirmation of the minima (see Text S1). The hypothetical TS structure was similar to the independently-postulated TSA, providing further support for 1,5-glucarolactone as an appropriate TSA. Density functional theory calculations were performed using a cluster model that included pNP-GLU and residues D163, E413, N466, R467, and E504. All calculations were run using Schrödinger Jaguar [54] with the hybrid B3LYP functional [55], [56] and 6-31G**+ basis set.

The TSA resembles the proposed TS (Figure 4B) through similar partial charges and stereochemistry within the carbohydrate moiety (see Figure 2). The TSA differs from the proposed TS by the replacement of the glycosidic bond with an ester functional group, resulting in an altered ring conformation due to the sp^2^-hybridized carbonyl. The differences between the TSA for pNP-GAL (i.e., 1,5-galactonolactone) and 1,5-glucarolactone are equivalent to the differences between pNP-GAL and pNP-GLU. These differences include changes in stereospecificity at the C4 carbon and the substituent at the C5 carbon (see Figure 2).

### Testing of TSA-based Redesign Paradigm Using k_cat_ and K_M_ Literature Data

Before proceeding with the redesign of GUS to accept the new substrate, we used existing k_cat_ and K_M_ data from literature to assess the validity of the proposed computationally-accessible metrics [48], [49], [57]. Earlier engineering efforts focused on altering GUS specificity from a substrate with a native carbohydrate topology (i.e., pNP-GLU) to a non-native one (i.e., pNP-GAL [49] or *para*-nitrophenyl- β, D-xylopyranoside [48]) or alternatively improving GUS resistance to glutaraldehyde and formaldehyde [57]. Therefore, the derived GUS mutants were less active towards pNP-GLU than wild-type (WT). We used the data to first assess whether the IE calculations at the ground state for the WT enzyme and a handful of available mutants were consistent with the experimental K_M_ values. We subsequently tested whether the reported k_cat_/K_M_ values were consistent with the IE calculations of the TSA with GUS.

The IE calculation included bond, angle, dihedral, improper dihedral, van der Waal, Urey-Bradley, electrostatic, NOE, and Generalized Born using Molecular Volume solvation energy terms under a single step CHARMM minimization. BE (Equation 1, where *G* is the Gibb’s free energy, *E·S* is the Michaelis complex, *E* is the unbound enzyme, *S* is the substrate, and *min* indicates that the structure is at the energy minimum) is here approximated by IE (Equation 2) for the enzyme-substrate complex.
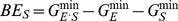
(1)

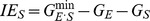
(2)


The entropic component of the free energy of solvation is accounted for by using an accessible area solvent model [58]. The change in solute entropy upon binding is assumed to be negligible relative to the other terms [59]. IE is a good surrogate for BE in cases where binding is not conditional on significant conformation rearrangements (no induced fit [60]). In addition, IE is less dependent on the force field as the energetics of any conformational rearrangements do not need to be tracked. IEs were calculated using the iterative protein redesign and optimization procedure (IPRO) [61]. IPRO iteratively randomly perturbs the protein backbone, subsequently assigns optimal rotamers for all design positions (mutable amino acid positions), and then executes an energy relaxation step. Different IPRO trajectories may converge in alternate low energy conformations. To remedy the run-dependent nature of the results, 25 separate IPRO trajectories were generated. The final IE was calculated as the average over the best IE for each one of the 25 trajectories (see Figures S2, S3, and S4 for distribution of IEs). In general, the energy distribution of the top 25 generated IEs followed trends that were consistent with a normal distribution. However, deviations away from a normal distribution are observed in some instances as a result of the small sample size. The calculated IE values were then related to K_M_ values obtained from literature as follows.

Michaelis-Menten kinetics for GUS enzymatic catalysis (based on the mechanism shown in Figure 4) is depicted through Equation 3, where *E* is GUS, *S* is pNP-GLU, *E·S* is GUS bound to pNP-GLU, *E·I_1_* is the bound carbocation intermediate, *E·I_2_* is the E504- covalent adduct, *E·P* is bound glucuronic acid, *P* is the product of the reaction (glucuronic acid), and *k* represents a reaction rate constant.
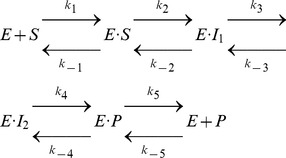
(3)


QM calculations *in vacuo* identified *E·S*, *E·I_1_,* and *E·I_2_* and found only a slight barrier between *E·I_1_* and *E·I_2_*. A TS for the *E·S* to *E·I_1_* step was not successfully located (see Text S1). Based on the QM calculations, it is unclear whether the rate-limiting step for GUS is *E·S* to *E·I_1_* or *E·I_2_* to *E·P*. However, both of these TSs are expected to closely resemble the carbocation intermediate (i.e., *E·I_1_*), which is consistent with the adopted TSA. By assuming a fast rate of hydrolysis of the covalent adduct (i.e., *E·I_2_*) and that the equilibrium constant of product release (i.e., *E+P*) after the rate-limiting step lies far to the right 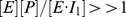, Equations 4 and 5 describe the enzyme catalytic parameters of the overall reaction (see Text S2 for detailed discussion of how these equations are arrived at from Equation 3).
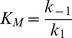
(4)

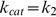
(5)



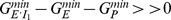
 is an alternate way of expressing that the equilibrium of product release lies far to the right. 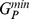 must be less than 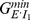. Otherwise, the intermediate would be the thermodynamically favored product, and an external energy source would be required to drive the reaction forward. Moreover, QM calculations (Table S1) inform us that the carbocation intermediate (i.e., *E·I_1_*) is a relatively high-energy intermediate. In addition, 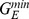 must assume a negative value for the enzyme to remain folded. Since, 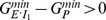 and 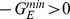, the equilibrium following the rate-limiting step must favor product release. The hypothetical rate-limiting step was used to identify the individual rate constants in Equations 4 and 5. However, the derivations are independent of the true rate-limiting step. The TSA choice does depend on the rate-limiting step, but it has been verified independently [52], [53].

Using the relationship between Gibb’s free energy and equilibrium concentrations (see Text S2, Equation S12), Equation 6 links the Michaelis constant, K_M_, to the BE between the enzymatic substrate complex (*E·S*) and the unbound reactants, BE_S_ (see Equation 1).
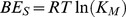
(6)


We find that the all-atom root mean square deviation (RMSD) between unbound (E) and bound (E*·*S) GUS is only 0.22 Å, implying that there is minimal conformational rearrangement in GUS upon binding [62] with pNP-GLU, which justifies the approximation of BE_S_ with IE_S_ (IE with the substrate, pNP-GLU) (see Equations 1 and 2). Using Equation 6 and the assumption that BE_S_ = IE_S_, we find that K_M_ and IE_s_ for the mutant/WT enzymes are related through Equation 7.
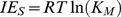
(7)


**Figure 5:**
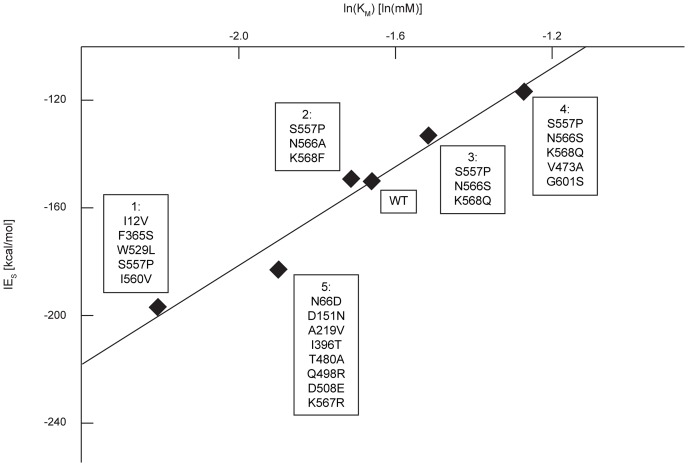
Ground state computational IE_S_ for pNP-GLU versus the natural logarithm of experimental K_M_. IEs were calculated using IPRO, and experimental data was obtained from literature [48], [49], [57]. Each numbered label corresponds to a single variant enzyme with multiple amino acid substitutions from wild-type (WT). Calculated IEs at the ground state are consistent with the observed changes in K_M_ for GUS mutants (R^2^ = 0.960). Figure S2 shows the distribution of the trajectory-best IEs whose average forms each data point.

Equation 7 implies a linear correlation between ln(K_M_) and IE_S_. Figure 5 depicts the measured K_M_ values [48], [49], [57] and corresponding calculated IE_S_s for the WT GUS and five mutants. The correlation coefficient of 0.960 implies that the derived expression (Equation 7) correctly captures the observed K_M_ trends for the enzyme variants. While the actual magnitude of the energy values on the y-axis is not quantitatively accurate, the relative ordering of the mutants in terms of their K_M_ values is consistent with the data.

**Figure 6:**
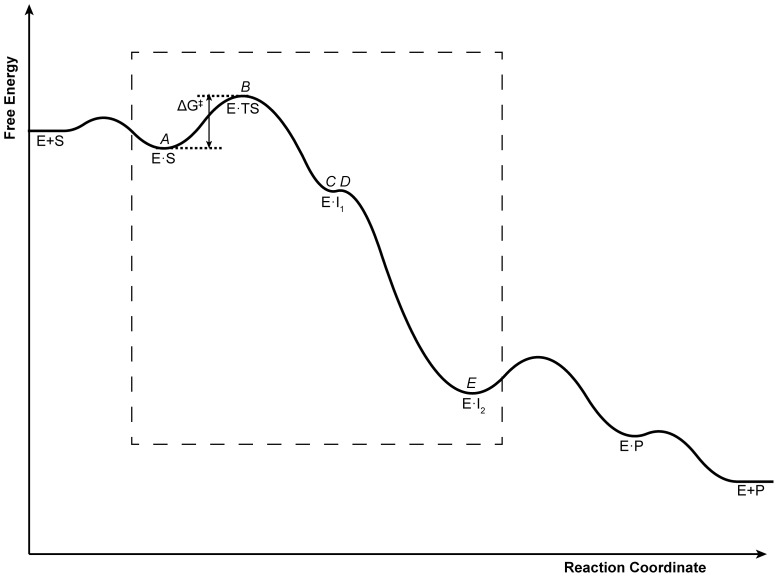
Qualitative GUS free energy diagram based upon *in vacuo* QM calculations. The free energy of each intermediate within the dashed box is based on its potential energy, as calculated using QM. Intermediates found using QM and proposed TSs are also labeled according to Figure 4 (*italicized, above curve*). The energy barrier between states *C* and *D* is nearly barrier-less. The free energy values along the remainder of the curve are purely hypothetical. Each intermediate is labeled according to the convention used in Equation 3. Based on the known and hypothesized free energies, the reaction of the Michaelis complex to form the first intermediate (k_2_, as written in Equation 3) is rate-limiting. Thus, the proposed TS for the entire reaction (E·TS) and its corresponding energy barrier (ΔG^‡^) are labeled.

Unlike K_M_, which depends on binding at the ground state, k_cat_ is directly related to the reaction rate. The rate constant of a reaction is related to the change in the Gibb’s free energy between the ground and TSs, based on the Eyring-Polanyi equation derived from transition state theory (Equation 8) (see also Figure 6).
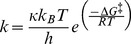
(8)


In Equation 8, *k* is the rate constant, *h* is Planck’s constant, κ is the transmission coefficient (assumed invariant among all mutants), *k_B_* is the Boltzmann constant, and Δ*G^‡^* is the change in Gibb’s free energy between the ground and TSs (Equation 9).
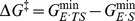
(9)


We cannot directly computationally assess Δ*G^‡^* because the TS structure is unknown. Since the structure of the TS is unavailable, we postulate *that mutations that lead to beneficial interactions of the enzyme with its TSA should produce similar benefits with the unresolved TS.* Equation 10 expresses this postulate mathematically by implying that the difference between the minimized free energy of the TS and the TSA is invariant with respect to mutations introduced on the enzyme.
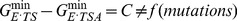
(10)


The unknown (for IPRO trajectories where the TSA is the ligand) free energy of the Michaelis complex can be eliminated by combining Equations 1 and 9, yielding Equation 11.
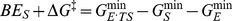
(11)


We have already shown that computationally approximated IE_S_ provides a good approximation for BE_s_. In analogy, we assume that the IE with the TSA (IE_TSA_) is a good approximation for BE_TSA_. The TSA and substrate structures, and therefore energies, remain largely unchanged during the redesign process. Since 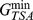 and 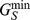 are both invariant with respect to mutations to the enzyme and IE_TSA_ ≅ BE_TSA_, Equation 10 can be used to eliminate the unknown free energy of the bound TS (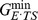) yielding Equation 12.
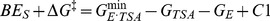
(12)


Constant *C1* is a grouping of constants, including those from Equations 8 and 10. Equation 12 is further simplified by substituting the definition of IE_TSA_ (see Equation 2, where the bound molecule in this case is the TSA).
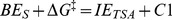
(13)


*C1* can be eliminated from Equation 13 by expressing it as the difference in the IEs between mutant and WT enzymes,
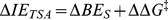
(14)where ΔIE_TSA_ = IE_TSA_−IE_TSA,WT_, ΔBE_S_ = BE_S_ – BE_S,WT_, and ΔΔ*G^‡^ = *Δ*G^‡^ −*Δ*G^‡^_WT_*. ΔBE_S_ and ΔΔ*G^‡^* can be recast using Equations 6 and 8 (at constant temperature).



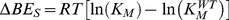
(15)

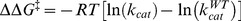
(16)


Substituting ΔBE_S_ from Equation 15 and ΔΔ*G^‡^* from Equation 16 into Equation 14 yields.
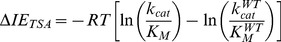
(17)


Equation 17 can be used to relate computationally accessible metrics to k_cat_/K_M_, which dictates the catalytic efficiency of the enzyme under substrate limiting conditions ([S]<<K_M_).

Equations 7 and 17 can be combined to directly link k_cat_ to computationally accessible metrics (Equation 18):
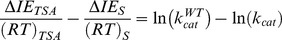
(18)


In Equation 18, ΔIE_S_ = IE_S_ – IE_S,WT_, *(RT)_TSA_* is the *RT* term in Equation 17, and *(RT)_S_* is the *RT* term in Equation 7. As an example, for GUS/pNP−GLU, *(RT)_TSA_* = 15.3 kJ/mol (T = 4.65 10^4^ K) while *(RT)_S_ = *386.7 kJ/mol (T = 1840 K). These temperature values were obtained through correlation analysis of Equations 17 and 7, respectively. Note that experimental and correlating temperatures do not match. Similarly high temperatures were seen in the quantification of RNA-ribosome binding calculations in the RBSCalculator [63].

**Figure 7:**
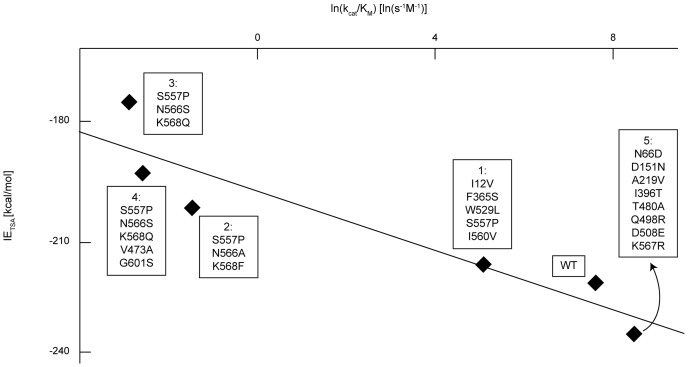
Computationally-determined IE_TSA_ for pNP-GLU versus experimental ln(k_cat_/K_M_). Data was collected as described in Figure 5. Enzyme variants with higher catalytic efficiency (k_cat_/K_M_) have a stronger affinity for 1,5-glucarolactone (R^2^ = 0.864). See also Figure S3.

**Figure 8:**
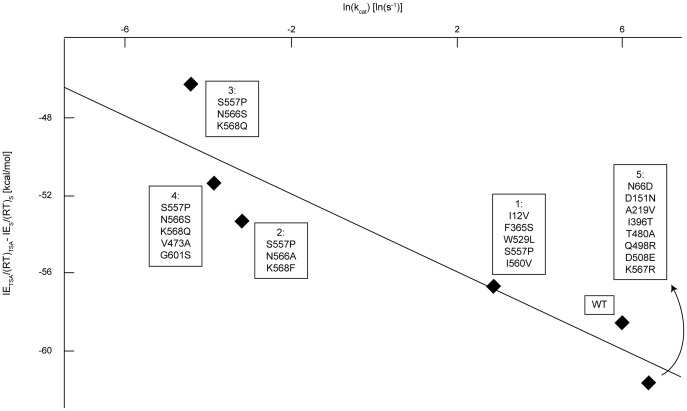
Scaled difference between IE_TSA_ and IE_S_ for pNP-GLU versus the natural logarithm of k_cat_. Data was obtained as detailed in the caption of Figure 5. Scaling is required because of the non-quantitative nature of the energy calculations. With scaling, it is apparent that the turnover number increases as the difference becomes more negative. These results suggest that as the enzyme interacts with the TS more strongly, the turnover number increases (R^2^ = 0.854).

**Figure 9:**
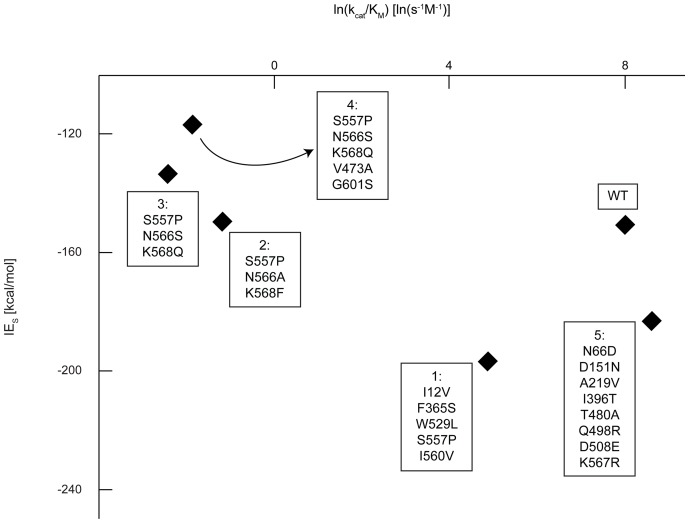
pNP-GLU IE_S_ correlation with catalytic efficiency. Data was obtained as described for Figure 5. No significant correlation is observed (R^2^ = 0.545) between IE with pNP-GLU and ln(k_cat_/K_M_). If GUS catalysis was primarily achieved through reactant destabilization, a positive slope would have been expected.

A strong correlation (R^2^ = 0.864) is observed between IE_TSA_ and the natural logarithm of experimental k_cat_/K_M_ values (see Figure 7), suggesting that IE_TSA_ is a good descriptor of k_cat_/K_M_. This observed correlation implies that the derived equations are applicable and that the chosen TSA is suitable. However, this trend does not necessarily prove the QM-based reaction mechanism. The same strong correlation (i.e., R^2^ = 0.854) is observed between IE_TSA_/(RT)_TSA_-IE_S_/(RT)_S_ and the natural logarithm of k_cat_ (see Figure 8). The experimental K_M_ values vary by less than an order of magnitude (Figure 5), while the experimental k_cat_/K_M_ values vary over several orders of magnitude (Figure 7). The scaling differences in the experimental data and the larger weight of 1/(RT)_TSA_ ( = 0.06 mol/kJ), relative to 1/(RT)_S_ ( = 0.002 mol/kJ), in the correlating expression (Equation 18) contribute to the similarity between Figure 7 and Figure 8. As a control, we verified that the energy difference between the Michaelis complex and unbound reactants shows no correlation with the catalytic efficiency (see Figure 9).

The justification of the chosen TSA and validation of the correlation between computationally-accessible metrics and experimental catalytic data justifies the use of IE calculations to optimize a targeted enzyme parameter.

## Results and Discussion

### Further Validation of Correlating Expressions Using pNP-GAL

Before implementing the OptZyme redesign approach, we first showed that the correlating expressions derived for pNP-GLU were transferrable to alternative substrates and their corresponding TSAs. Since our overarching goal was to switch GUS specificity from pNP-GLU to pNP-GAL, we sought to verify the correlating expressions for K_M_ (Equation 7) and k_cat_/K_M_ (Equation 17) using pNP-GAL and 1,5-galactonolactone, respectively. pNP-GAL k_cat_/K_M_ data was again obtained from literature sources focused on altering GUS specificity from pNP-GLU to pNP-GAL [47], [49]. Accurate K_M_ estimates were absent in the literature. Instead, we estimated them by monitoring *para*-nitrophenolate absorbance as a function of substrate concentration and fitting to the Michaelis-Menten equation using the mutant cell lysates (see Text S3). The K_M_ value determined for the native substrate analogue (i.e., pNP-GLU) using the same crude lysate of WT GUS (0.242±0.022 mM) was similar to the literature reported value (0.183 mM [48], [49], [57]).

**Figure 10:**
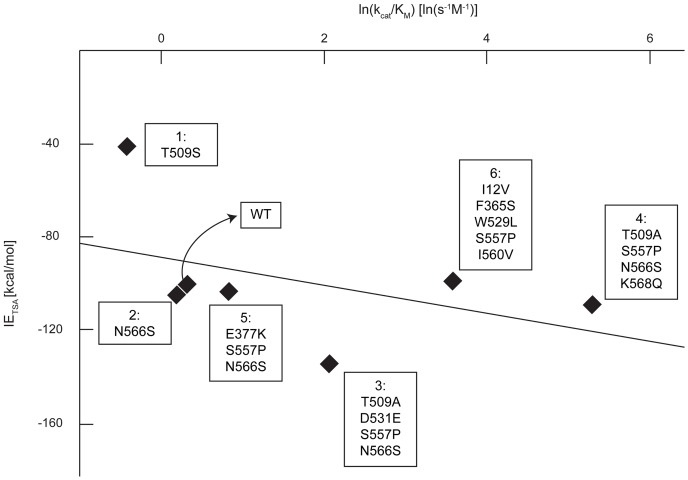
Correlation between pNP-GAL IE_TSA_ and ln(k_cat_/K_M_). The correlation found here is significantly lower than the one found for pNP-GLU (see Figure 7) primarily due to mutant *T509S*. See also Figure S4.

The observed k_cat_/K_M_ correlation for pNP-GAL (Figure 10, Equation 17) was similar (albeit weaker) to that for pNP-GLU (see Figure 7), with the exception of one outlier (i.e., *T509S*). The observed K_M_ correlation for pNP-GAL (Equation 7) has a positive slope, similar to the correlation for pNP-GLU (see Figure 5). However, one of the three variants (i.e., *T509A*, *D531E*, *S557P*, *N566S*) was an outlier. Considering both pNP-GLU and pNP-GAL mutant data, *D531E* was the only surface point mutation located near the center of an α-helix. Implicit solvation models have been shown to cause inaccuracies within α-helices [64]. By considering pNP-GAL, we demonstrated the applicability of Equations 7 and 17 of OptZyme for non-native substrates.

### Redesign of GUS for Improving Activity with pNP-GLU

OptZyme was first used to identify beneficial mutations that improve K_M_, k_cat_/K_M_, and k_cat_ with pNP-GLU by minimizing the appropriate IE (Equations 7, 17 and 18, respectively). Constraints that ensure that both the substrate and TSA favorably bind GUS (i.e., IE_S_<0, IE_TSA_<0) were included in the OptZyme runs. Design positions were selected in locations that are likely to impact active site geometry and directly mediate interactions with the substrate. The same set of design positions was chosen for all sets of calculations (H162, D163, F164, V355, G356, L361, G362, W549, N550).

**Figure 11:**
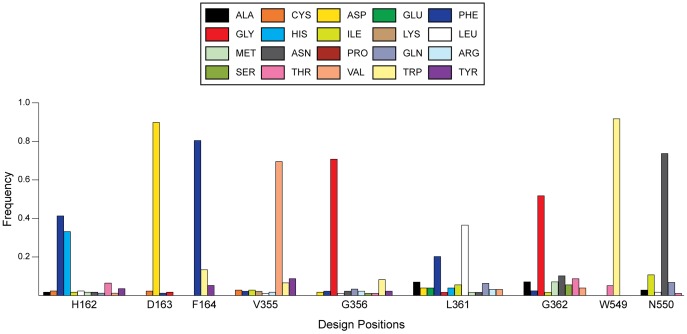
Distribution of amino acids in a sequence alignment for all β-glucuronidases. The sequence alignment was performed over all β-glucuronidases (as identified using BRENDA) using the Clustal-Omega algorithm. 181 unique sequences were used during the alignment. Design position numbers indicate the position within GUS, and the one-letter abbreviation for WT *E. coli* β-glucuronidase is provided at each position. Only amino acids observed >1% of the time at a given position are shown since smaller bars were difficult to decipher. With the exception of H162, the *E.* *coli* WT residue is the amino acid most frequently observed in the alignment.

A high frequency of mutations to glycine by OptZyme was initially observed, presumably to avoid steric clashes within the highly-packed active site of GUS. To remedy this bias, we first performed multiple sequence alignments to extract natural amino acid usage patterns. The first family alignment was performed using PFAM [65] between GUS and the glycosyl hydrolases family 2, and the second alignment was performed between GUS and all other β-glucuronidases (as identified in BRENDA [66]) using Clustal-Omega [67]. Amino acids observed at least once in the alignment of all β-glucuronidases (181 sequences, see Figure 11) or in at least 5% of the glycosyl hydrolases family 2 (excluding gaps, 3975 sequences) were permitted for each design position (see Table 2 for permissible mutations). In addition, the total number of glycine residues throughout all design positions was restricted to be at most two (matching the glycine utilization frequency in WT).

**Table 2:** Permitted amino acids at each design position.

Design Position	Permitted Amino Acids
162	A, C, F, G, H, I, L, M, N, Q, S, T, V, Y
163	C, D, F, G, K, M, Q, R, S, T, W
164	F, M, Q, W, Y
355	A, C, E, F, H, I, K, L, M, R, V, W, Y
356	A, C, D, E, F, G, H, I, L, M, N, Q, R, S, T, W, Y
361	A, D, E, F, G, H, I, K, L, M, N, Q, R, S, T, V, Y
362	A, D, F, G, I, K, M, N, R, S, T, V
549	A, C, G, K, L, R, T, W, Y
550	A, E, F, G, I, L, N, Q, S, T, V, Y

This table contains the list of permitted amino acids (using one-letter abbreviations) at each design position. Amino acids were permitted if they appeared at least once in the β-glucuronidase alignment or observed in at least 5% of the glycosyl hydrolases family 2.

Fifty independent trajectories of OptZyme were run to optimize K_M_, k_cat_/K_M_, and k_cat_ for GUS using pNP-GLU and 1,5-glucarolactone. NOE restraints were used to maintain the optimal catalytic geometry of GUS (Table 1, Figure 3). Each trajectory of OptZyme consisted of 5000 iterations, and simulated annealing was used after 100 cycles (using constant T = 7268K, which corresponds to an acceptance rate of about 50% of redesigns within 10 kcal/mol, 41.9 kJ/mol, of the best mutant) to avoid premature convergence to local minima of the GUS free energy landscape. The CHARMM energy terms used were identical to those used in the testing of the TSA-based redesign paradigm, and the backbone-dependent Dunbrack rotamer library was used for side chain optimization [68].

**Figure 12:**
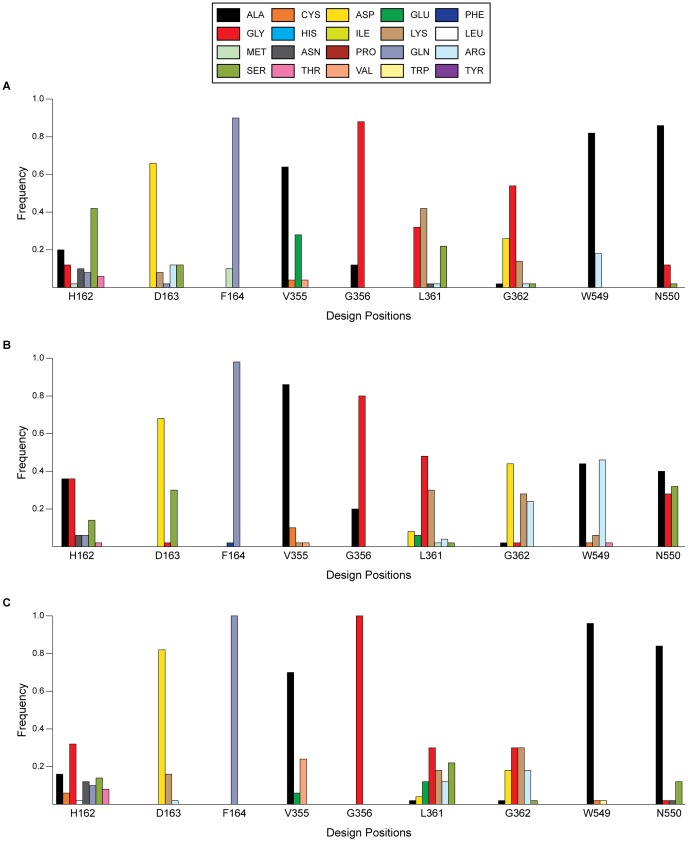
Distribution of amino acids for top 50 GUS mutants enhancing enzyme catalytic parameters of pNP-GLU. The libraries were designed to optimize (*A*) K_M_, (*B*), k_cat_/K_M_, and (*C*) k_cat_. Design position numbers indicate the position within GUS, and the one-letter abbreviation for WT GUS is provided.

OptZyme was used to identify three libraries of mutants that were computationally predicted to enhance enzyme catalytic parameters relative to WT (see Table 3, Figure 12). The observed mutants seemed to lower the relevant IE predominantly through improving flexibility in the active site, increasing solvation stabilization, or improving the electrostatic IE (including hydrogen bonding). Many mutations were common between the K_M_- and k_cat_/K_M_-optimized libraries because of the electrostatic and structural similarity between the substrate and TSA. In the interest of identifying mutations that primarily improve a specific enzyme parameter, a systematic cutoff was defined for identifying mutations that were representative of the K_M_- or k_cat_/K_M_- optimized libraries. A mutation was considered representative of a library if it occurred at least 15% of the time for a given design position and at the same time 10% more frequently than in the other libraries. These metrics were selected because they closely matched the representative mutations determined by visual inspection of Figure 12. For example, *H162A* and *H162G* (extra flexibility of the protein backbone), *D163S* (enhanced solvation), and *G362R* (hydrogen bonding/solvation effects) were mutations representative of the pNP-GLU k_cat_/K_M_-optimized library (see Figure 12).

**Table 3:** Top 10 mutants identified using OptZyme for optimizing K_M_, k_cat_/K_M_, and k_cat_ for pNP-GLU.

			Design Positions								
Parameter	Rank	Energy	162	163	164	355	356	361	362	549	550
**K_M_**	WT	−489.4	H	D	F	V	G	L	G	W	N
	1	−1548.7	Q	D	Q	A	A	G	D	R	G
	2	−1513.6	A	D	Q	A	G	G	R	R	A
	3	−1509.8	G	S	Q	A	G	K	D	A	S
	4	−1482.6	Q	D	Q	A	A	G	D	A	G
	5	−1477.6	A	S	Q	A	G	G	D	A	A
	6	−1455.9	S	S	Q	A	A	G	D	A	G
	7	−1454.6	A	S	F	A	A	G	D	A	G
	8	−1440.0	A	S	Q	A	G	G	R	A	S
	9	−1434.1	S	D	Q	A	G	G	R	R	A
	10	−1429.9	A	S	Q	C	G	K	D	A	G
**k_cat_/K_M_**	WT	−377.8	H	D	F	V	G	L	G	W	N
	1	−1570.6	N	D	Q	A	G	G	D	A	A
	2	−1561.4	N	D	Q	A	G	G	K	A	A
	3	−1560.2	A	D	Q	A	G	G	K	A	A
	4	−1551.4	G	K	Q	A	G	S	K	A	A
	5	−1531.3	A	R	Q	A	G	S	G	A	A
	6	−1520.8	Q	D	Q	A	G	G	D	A	A
	7	−1518.3	A	D	Q	A	G	K	D	A	G
	8	−1514.2	A	D	Q	A	G	G	D	A	A
	9	−1502.9	S	D	Q	A	G	G	S	A	A
	10	−1495.3	S	S	Q	E	G	K	G	A	A
**k_cat_**	WT	−129.7	H	D	F	V	G	L	G	W	N
	1	−380.6	G	D	Q	A	G	R	D	A	A
	2	−375.1	G	D	Q	A	G	K	D	A	A
	3	−374.5	Q	D	Q	A	G	G	R	A	A
	4	−361.0	G	D	Q	A	G	E	K	A	A
	5	−355.5	N	D	Q	V	G	S	G	A	A
	6	−349.3	C	D	Q	V	G	S	G	A	A
	7	−346.7	G	K	Q	E	G	S	R	A	A
	8	−341.3	G	K	Q	A	G	A	K	A	A
	9	−340.8	Q	D	Q	A	G	G	K	A	A
	10	−336.1	L	D	Q	A	G	G	K	A	A

One-letter amino acid abbreviations for each design position and WT residue. Energy values are in kJ/mol.

Experimental validation of the mutants can be carried out using a high-throughput assay, where the fluorescence of the *para*-nitrophenolate leaving group is readily measured based on its high absorbance at 405 nm [47]. The design of mutants for pNP-GLU is handicapped as WT GUS is already very active and the scope for identifying significantly improved mutants is limited. However, GUS activity with pNP-GAL is ∼10^7^ lower than for pNP-GLU [47]. Therefore, the entire gamut of beneficial interactions leading to switch of specificity from pNP-GLU to pNP-GAL would be detectable using a high-throughput assay.

### Redesign of GUS for Introducing Catalytic Activity with the New Substrate pNP-GAL

**Figure 13:**
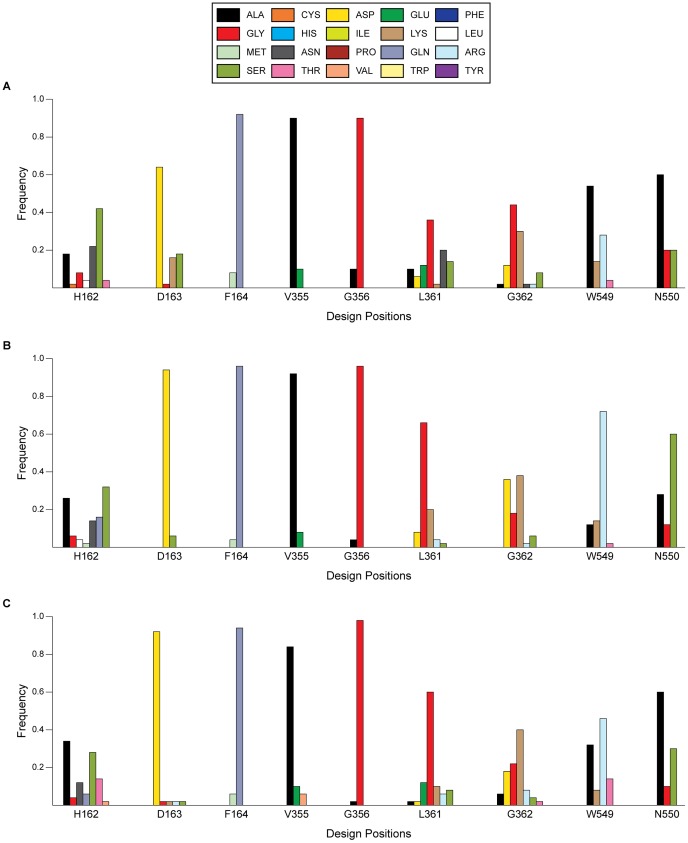
Distribution of amino acids for top 50 GUS mutants enhancing enzyme catalytic parameters of pNP-GAL. The libraries were designed to optimize (*A*) K_M_, (*B*), k_cat_/K_M_, and (*C*) k_cat_. Design position numbers indicate the position within GUS, and the one-letter abbreviation for WT GUS is provided.

Three libraries were constructed that were designed to enhance K_M_, k_cat_/K_M_, or k_cat_ of GUS for pNP-GAL (see Table 4, Figure 13). The constructed mutants were stabilized in a similar manner as described for pNP-GLU. The only representative mutant in the pNP-GAL K_M_-optimized library was *L361N* (electrostatic interactions with pNP-GAL C5 substituent/solvation enhancement). *L361G* (extra flexibility of GUS backbone), *W549R* (hydrogen bonding with pNP-GAL C2 hydroxyl group), and *N550S* (solvation enhancement) were representative mutants for the pNP-GAL k_cat_/K_M_-optimized library.

**Table 4:** Top 10 mutants identified using OptZyme for optimizing K_M_, k_cat_/K_M_, and k_cat_ for pNP-GAL.

			Design Positions								
Parameter	Rank	Energy	162	163	164	355	356	361	362	549	550
**K_M_**	WT	−70.7	H	D	F	V	G	L	G	W	N
	1	−1528.8	S	D	Q	A	G	G	K	A	A
	2	−1514.2	S	D	Q	A	G	N	G	R	S
	3	−1505.0	S	K	Q	A	G	E	K	A	G
	4	−1472.3	A	K	Q	A	A	S	G	A	G
	5	−1453.1	N	D	Q	A	G	N	G	R	S
	6	−1442.6	S	S	Q	A	G	A	G	A	A
	7	−1435.5	S	D	Q	A	G	D	K	K	G
	8	−1423.8	S	S	Q	A	G	G	D	A	A
	9	−1413.7	S	D	Q	A	G	N	G	K	S
	10	−1408.7	G	K	Q	E	G	A	A	A	A
**k_cat_/K_M_**	WT	90.1	H	D	F	V	G	L	G	W	N
	1	−1041.2	A	D	Q	A	G	R	G	R	S
	2	−1034.9	Q	D	Q	A	G	K	D	R	G
	3	−1004.4	S	D	Q	A	G	G	S	R	S
	4	−975.1	A	D	Q	A	G	G	S	R	S
	5	−969.7	N	D	Q	A	G	G	D	K	S
	6	−956.7	N	D	Q	A	G	G	D	R	S
	7	−940.8	S	D	Q	A	G	G	K	R	S
	8	−931.2	Q	D	Q	A	G	D	K	A	G
	9	−930.3	A	D	Q	A	G	G	D	K	S
	10	−924.9	A	D	Q	A	G	G	K	K	S
**k_cat_**	WT	25.4	H	D	F	V	G	L	G	W	N
	1	−251.9	G	D	Q	A	G	E	K	A	A
	2	−245.3	A	D	Q	A	G	G	R	T	A
	3	−244.2	A	D	Q	A	G	G	A	T	A
	4	−240.0	A	D	Q	A	G	G	K	T	A
	5	−234.2	A	D	Q	A	G	E	K	A	A
	6	−233.7	S	D	Q	A	G	D	K	A	G
	7	−232.7	A	D	Q	A	G	G	K	K	S
	8	−232.5	A	D	M	A	G	G	S	R	S
	9	−227.9	G	D	Q	A	G	E	K	R	S
	10	−220.3	S	D	Q	A	G	S	G	K	S

One-letter amino acid abbreviations for each design position and WT residue. Energy values are in kJ/mol.

Mutations enriched in the pNP-GAL libraries but largely absent from all pNP-GLU libraries were also identified. The analysis revealed only one such additional mutation, *H162N* (electrostatic interaction with the C4 substituent). Structural analysis also revealed that the backbone carbonyl of F161 (not a design position) formed a hydrogen bond in 97.5% of the examined structures (each mutant in Table 4) with the C5 substituent of pNP-GAL. This interaction was absent for all of the pNP-GLU designs in Table 3. Thus, the identity of the adjacent residue at design position 162 may directly promote (or prevent) the backbone interaction with pNP-GAL. In addition, mutation *H162S* is observed in 13.3% of the examined mutants (see Table 3) for pNP-GLU but 36.7% (see Table 4) for the pNP-GAL libraries. Therefore, *H162S* may be important for the interaction of F161 with pNP-GAL.

Several mutations were found that make direct contact with the novel ligand. Since the differences between pNP-GLU and pNP-GAL are in the C4 and C5 substituents of the carbohydrate moiety (Figure 2), mutations that create contacts with these substituents are expected. Indeed, this is the case for the *D163K*, *L361R*, and *L361E* mutations, as well as the contact by F161. However, *W549R* forms a contact with the ligand but at the unchanged portion of the carbohydrate. *W549R* was more common in the pNP-GAL libraries because of a slightly deformed sugar ring of pNP-GAL, relative to pNP-GLU. The results show OptZyme is sensitive enough to detect even minor structural variances between substrates.

Amongst the pNP-GAL libraries, the K_M_-optimized library is enriched with smaller amino acids (see Text S4 for discussion on prevalence of small amino acids). Although this observation could be an artifact due to the larger size of pNP-GAL relative to its TSA, the design positions were chosen at the edge of the active site further away from the pNP substituent. Thus, the smaller side chains in the K_M_-optimized library are more likely a reflection of the chair-like conformation of the sugar ring, which has a larger excluded volume than the planar geometry of the TSA. The mutation of the WT side chains to the large, polarizable side chains that are representative of the k_cat_/K_M_-optimized library (*H162Q*, *L361K*, *G362D*, *W549R*), imply that the planar form of the molecule is stabilized through efficient packing of the enzyme and beneficial electrostatic interactions.

### Summary

A new set of computationally accessible metrics was derived for correlating K_M_, k_cat_/K_M_, and k_cat_ between WT and mutated enzymes. With the aid of a QM-derived reaction mechanism, we validated that the IE_S_ correlates with K_M_ (Equation 7), and the IE_TSA_ correlates with k_cat_/K_M_ (Equation 17). k_cat_ can be measured through a weighted combination of IE_S_ and IE_TSA_ (Equation 18). It is important to note that the observed correlations are not proof for the QM-based mechanism. OptZyme, a computational tool used to design mutations that improve K_M_, k_cat_, or k_cat_/K_M_, generated mutations that were predicted to enhance enzymatic activity for pNP-GLU. OptZyme is best suited for systems where the solute entropy change upon binding is assumed to be negligible relative to other terms, substrate binding is not a consequence of “induced fit”, and equilibrium following the rate-limiting step strongly favors product release. The identified mutants stabilized the substrate mostly through hydrogen bonding networks, improved solvation, and efficient packing of the active site. OptZyme was utilized to construct a library of mutants with improved enzyme catalytic parameters for a similar substrate, pNP-GAL. Though these substrates are similar, OptZyme was able to identify novel contacts with the ligand in the pNP-GAL libraries that were absent from the pNP-GLU libraries. Several mutations were enriched in all of the pNP-GAL libraries, namely those that interact with the distorted sugar ring conformation or its altered substituents. In comparison of the K_M_- and k_cat_/K_M_-optimized libraries for pNP-GAL, we found that large, polar side chains were observed more often in the k_cat_/K_M_-optimized library. This was attributed to the more planar geometry of the TSA. These results suggest that mutants with large, polar side chains can stabilize the TS through interactions with the hydroxyl substituents and efficient packing, thereby improving enzymatic activity. OptZyme is available for download at maranas.che.psu.edu/submission/OptZyme.htm.

## Supporting Information

Figure S1 **Dihedral angles of ground state and TSA for pNP-GLU and pNP-GAL.** The layout of this figure corresponds to the layout of Figure 2. TS dihedral angles could not be determined because the TS structure was never solved so its coordinates are unknown. Dihedral angles were calculated using only the six atoms constituting the sugar ring (five carbon atoms, one oxygen atom). The absolute value of the dihedral angles describing the rotation about the C6-O, C1-O, and C1–C2 bonds are much lower for the TSAs than for the ground state molecules. This illustrates the more planar ring geometry of the TSAs.
(TIF)

Figure S2 **Distribution of individual pNP-GLU IE_S_ values.** The bins within the histogram were formed according to Doane’s formula (Doane, 1976). A normal distribution was included to compare against the computational data. The normal distribution was constructed by calculating the mean and standard deviation over the 25 individual values. The mean of the 25 values was used in Figure 5.
(TIF)

Figure S3 **Variance of individual pNP-GLU IE_TSA_ values.** The figure was generated as described for Figure S2. The mean of the 25 separate values was incorporated into Figure 7.
(TIF)

Figure S4 **Distribution of pNP-GAL IE_TSA_ values.** The figure was constructed as described for Figure S2. The average over the 25 individual IE_TSA_ values was used within Figure 10.
(TIF)

Figure S5 **pNP-GAL K_M_ Estimation for GUS R2 Variant.** The K_M_ value was determined by fitting to the Michaelis-Menten equation using nonlinear regression analysis. The data was collected for the fitting procedure by monitoring pNP absorbance as a function of substrate concentration in the cell lysate. For the GUS R2 mutant using pNP-GAL as the substrate, K_M_ = 25.4±0.3 mM (R^2^ = 0.999).
(TIF)

Figure S6 **pNP-GAL K_M_ Approximation for GUS R2.8 Variant.** The fitting procedure is identical to that described for Figure S5. For the GUS R2.8 variant using pNP-GAL as the substrate, K_M_ = 29.0±2.7 mM (R^2^ = 0.998).
(TIF)

Figure S7 **Ramachandran plot of top pNP-GAL mutants.** 50 of the top mutants from each of the pNP-GAL libraries were examined. “Core” (white), “allowed” (off white), “generous” (gray), and “outside” (dark gray) regions of the Ramachandran plot were determined by Morris *et al.* (1992). Results show that glycine residues (crosses) are frequently observed in the “generous” or “outside” regions of the map. Alternatively, the other 19 standard amino acids (squares) are much less frequently observed in the “generous” or “outside” regions. Glycine residues can avoid some of the steric repulsion that is more difficult to avoid for residues with a C_β_. While other amino acids can undergo contortions in their side chain to avoid a strong steric clash, mutation to a glycine residue is more favorable.
(TIF)

Table S1 **Gas phase energies from QM cluster model of GUS active site.** The gas phase energies are reported for the cluster model of the active site with the backbone of all residues constrained, as well as the ASN 466 sidechain. The calculated energies are relative to the calculated “Intermediate 2 (E)” energy. Each of the three structures corresponds to structures identified in Figures 4 and 6. This correspondence is indicated by each structure’s one-letter label.
(DOC)

Table S2 **Primers Used for Switching GUS Specificity.**
(DOC)

Text S1 **Detailed Discussion of QM Calculations.**
(DOC)

Text S2 **Derivation of** **Equations 4** **&** **5** **.**
(DOC)

Text S3 **Experimental Methods for K_M_ Estimation from Cell Lysates.**
(DOC)

Text S4 **Prevalence of Small Amino Acids in OptZyme Results.**
(DOC)
