# Supplementary figures and images for: OptZyme: Computational Enzyme Redesign Using Transition State Analogues
Source: PLoS One. 2013 Oct 7;8(10):e75358. doi: 10.1371/journal.pone.0075358 (PMC3792102; doi:10.1371/journal.pone.0075358)

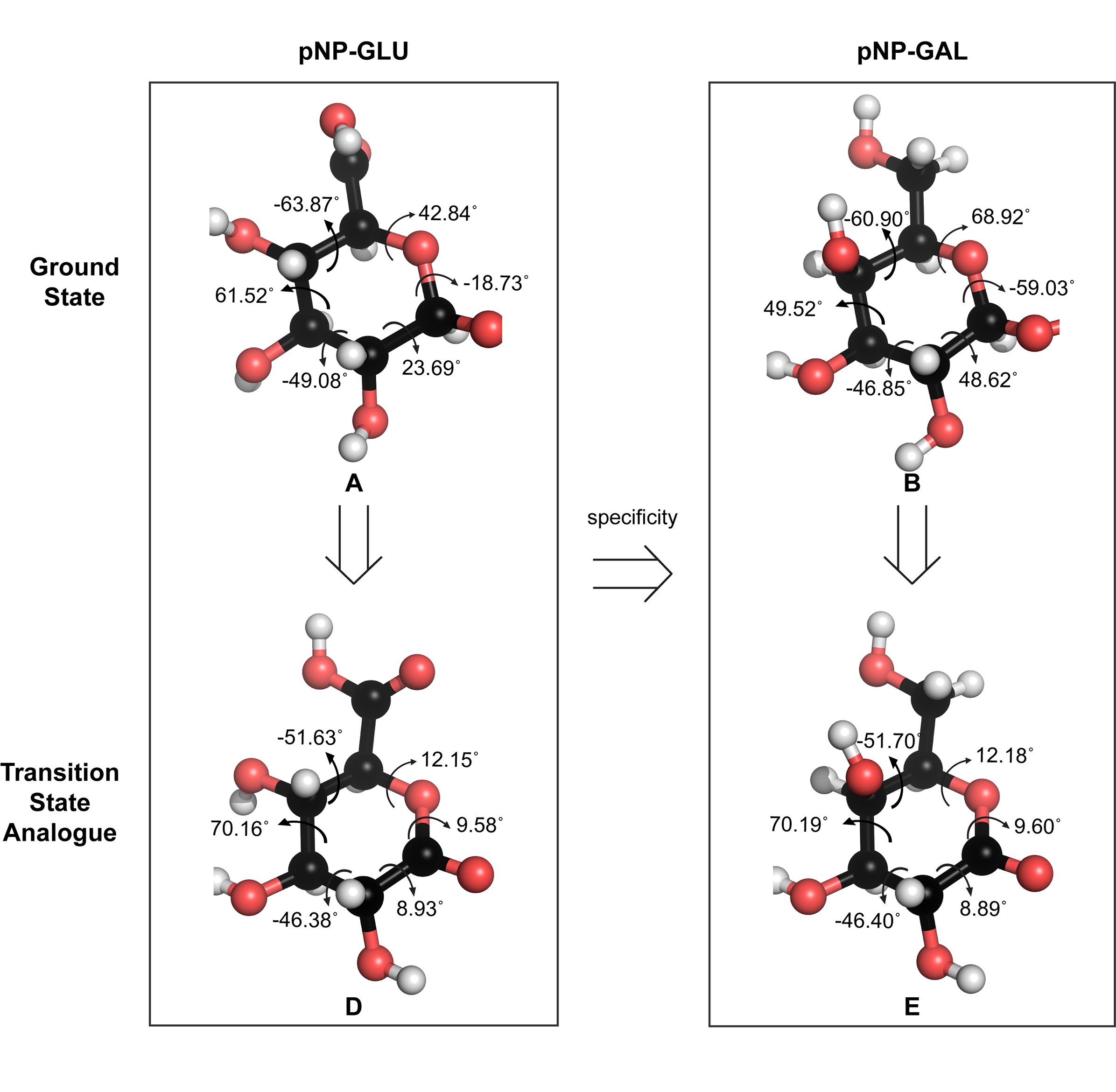

Supplement: Figure S1 — Dihedral angles of ground state and TSA for pNP-GLU and pNP-GAL. The layout of this figure corresponds to the layout of Figure 2. TS dihedral angles could not be determined because the TS structure was never solved so its coordinates are unknown. Dihedral angles were calculated using only the six atoms constituting the sugar ring (five carbon atoms, one oxygen atom). The absolute value of the dihedral angles describing the rotation about the C6-O, C1-O, and C1–C2 bonds are much lower for the TSAs than for the ground state molecules. This illustrates the more planar ring geometry of the TSAs. (TIF) [file pone.0075358.s001.tif]

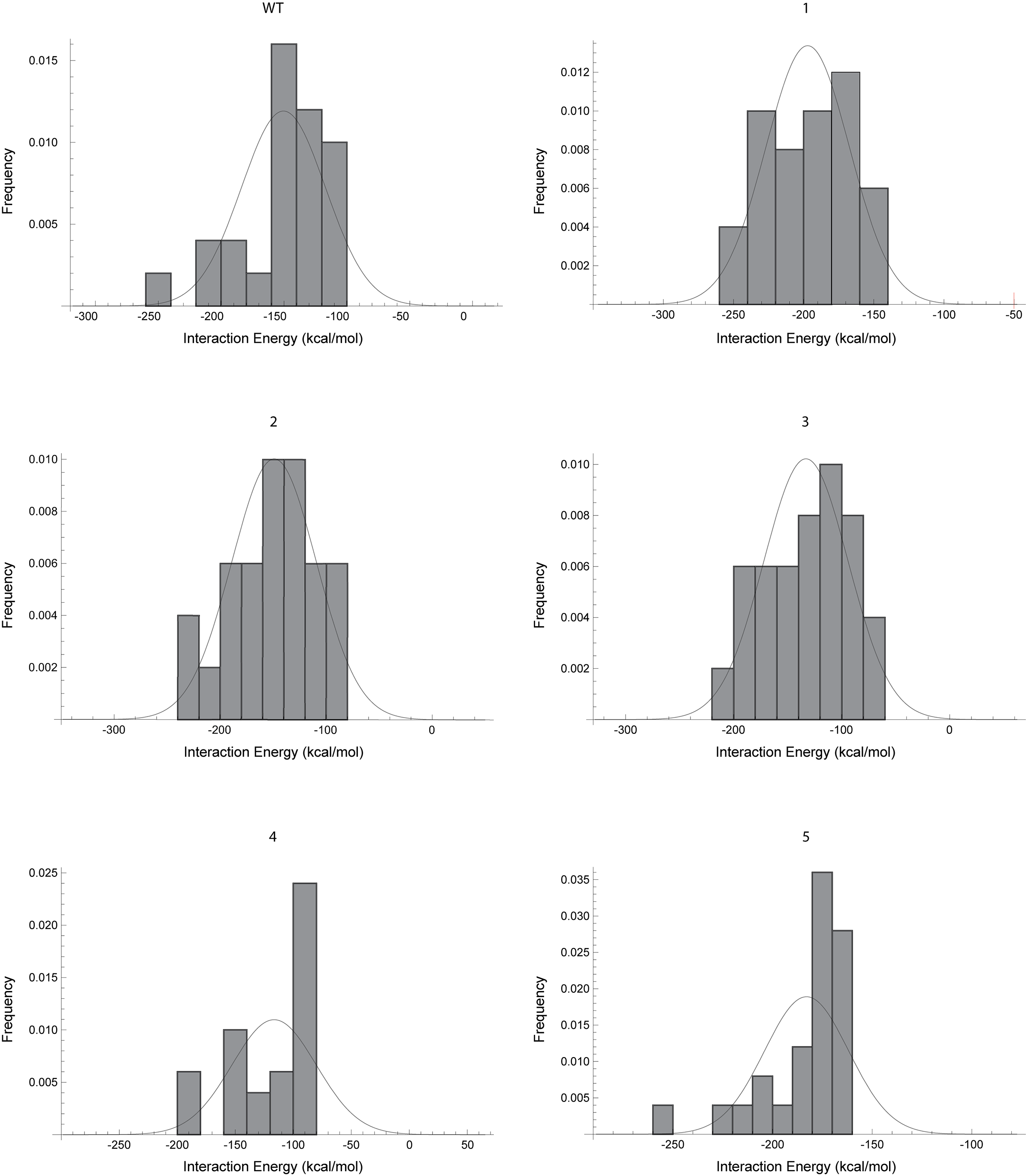

Supplement: Figure S2 — Distribution of individual pNP-GLU IES values. The bins within the histogram were formed according to Doane’s formula (Doane, 1976). A normal distribution was included to compare against the computational data. The normal distribution was constructed by calculating the mean and standard deviation over the 25 individual values. The mean of the 25 values was used in Figure 5. (TIF) [file pone.0075358.s002.tif]

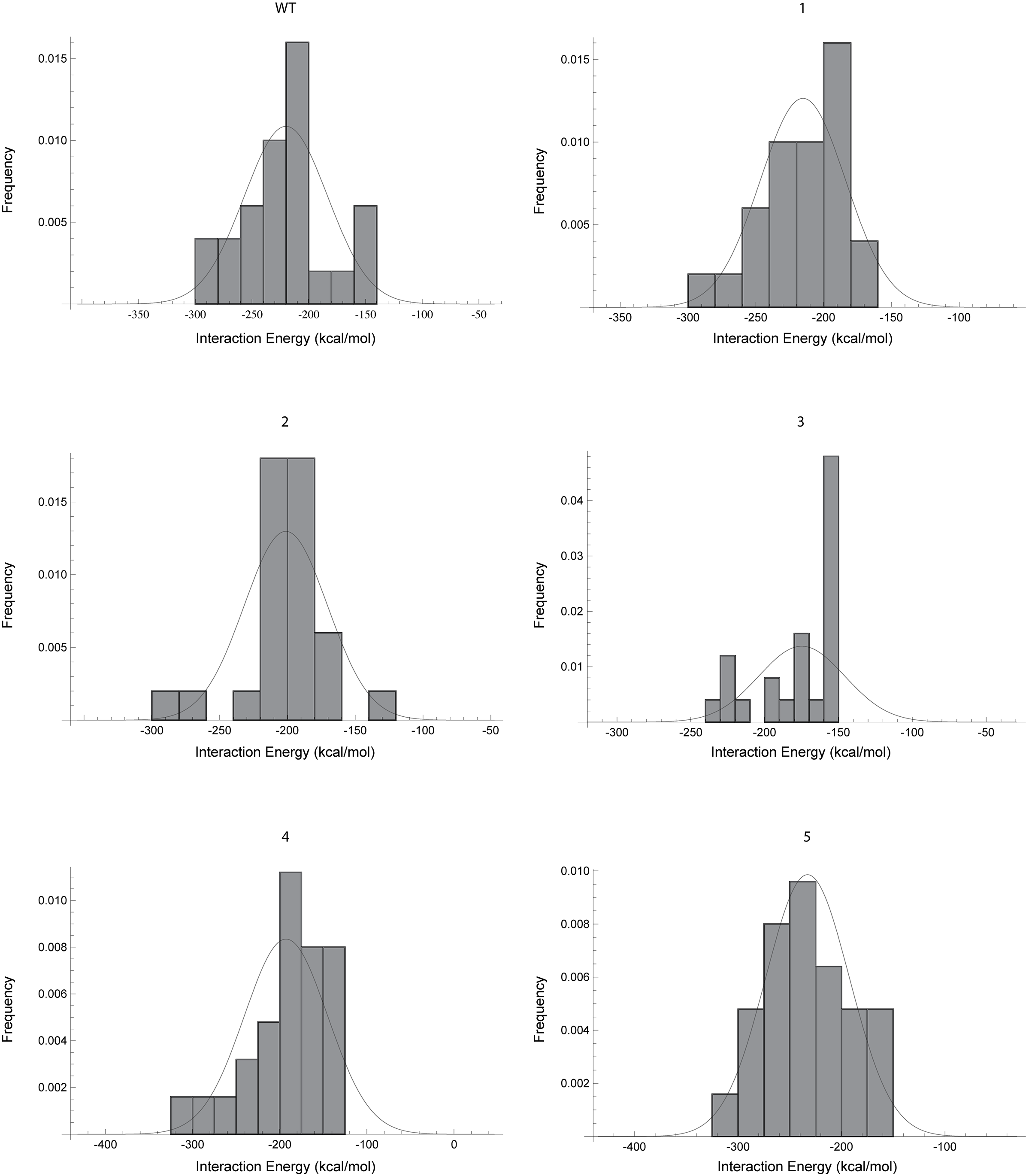

Supplement: Figure S3 — Variance of individual pNP-GLU IETSA values. The figure was generated as described for Figure S2. The mean of the 25 separate values was incorporated into Figure 7. (TIF) [file pone.0075358.s003.tif]

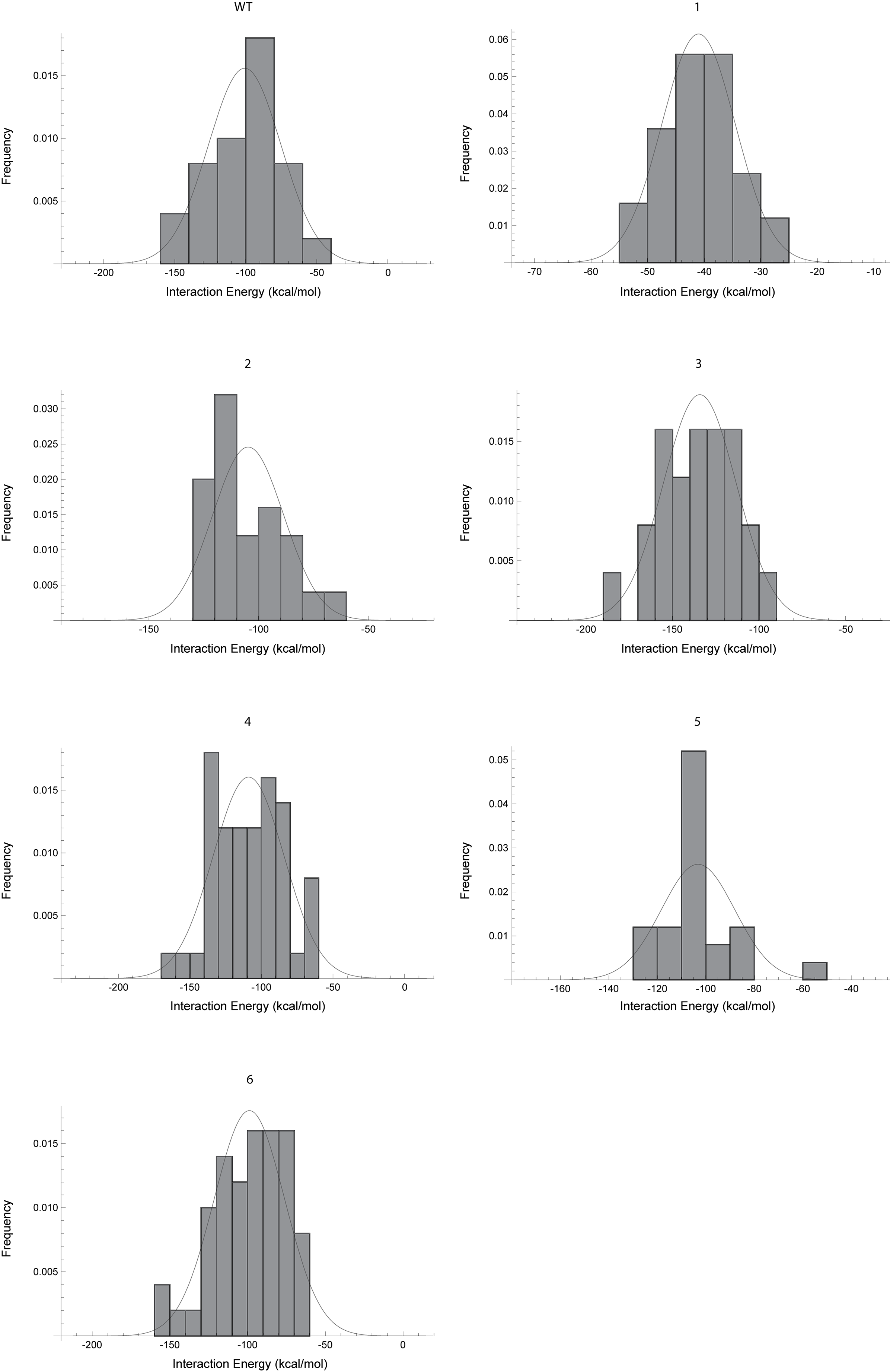

Supplement: Figure S4 — Distribution of pNP-GAL IETSA values. The figure was constructed as described for Figure S2. The average over the 25 individual IETSA values was used within Figure 10. (TIF) [file pone.0075358.s004.tif]

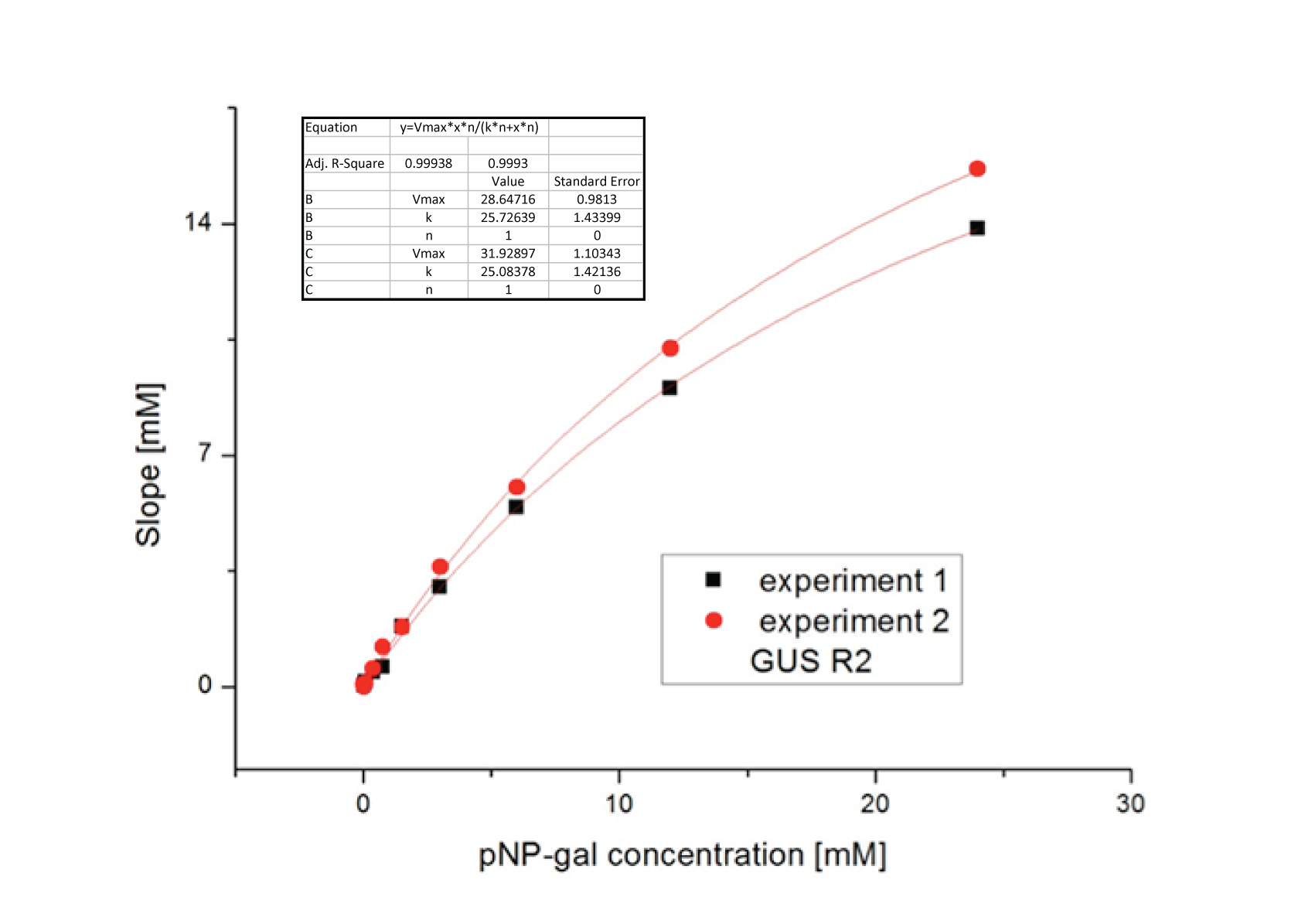

Supplement: Figure S5 — pNP-GAL KM Estimation for GUS R2 Variant. The KM value was determined by fitting to the Michaelis-Menten equation using nonlinear regression analysis. The data was collected for the fitting procedure by monitoring pNP absorbance as a function of substrate concentration in the cell lysate. For the GUS R2 mutant using pNP-GAL as the substrate, KM = 25.4±0.3 mM (R2 = 0.999). (TIF) [file pone.0075358.s005.tif]

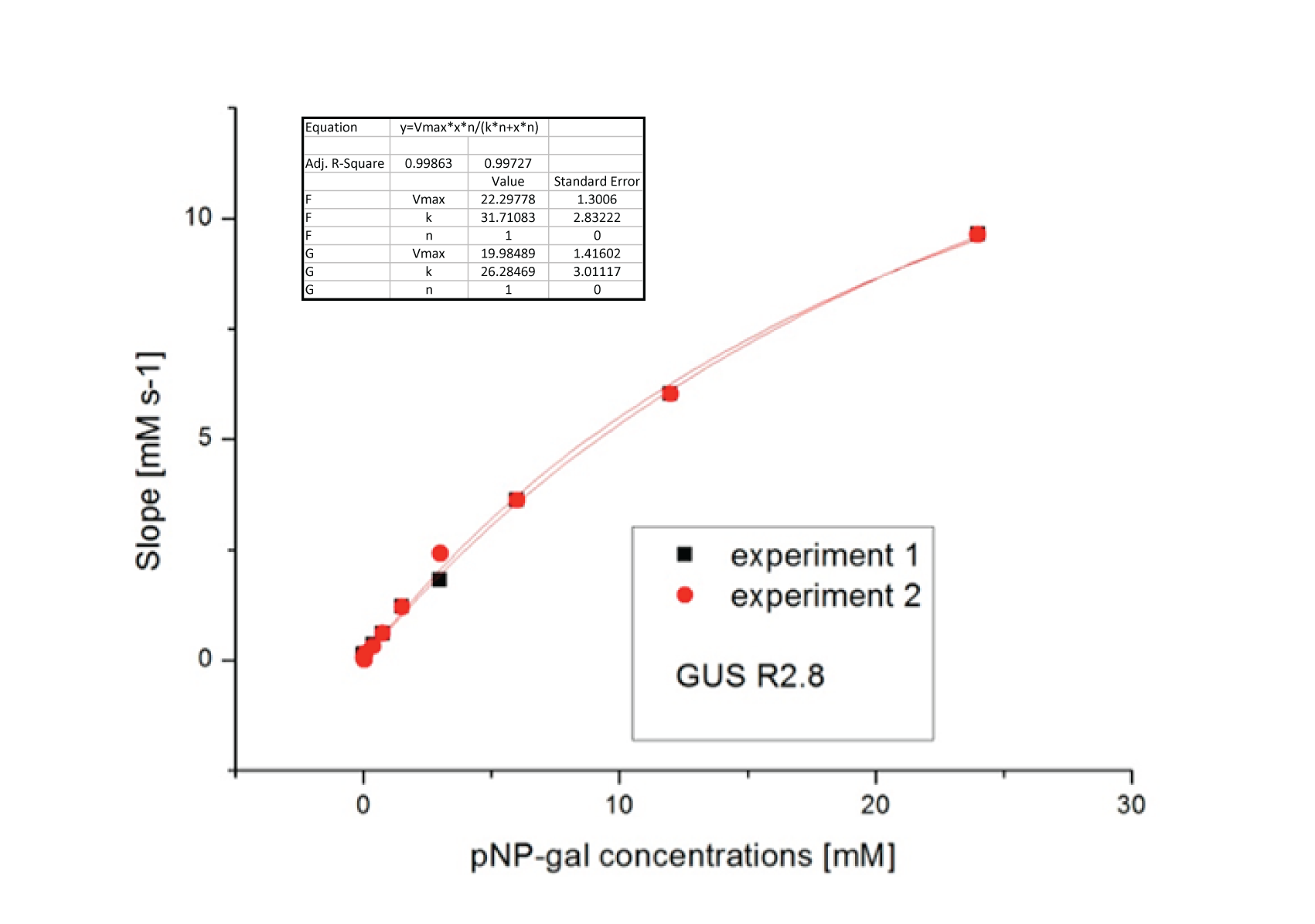

Supplement: Figure S6 — pNP-GAL KM Approximation for GUS R2.8 Variant. The fitting procedure is identical to that described for Figure S5. For the GUS R2.8 variant using pNP-GAL as the substrate, KM = 29.0±2.7 mM (R2 = 0.998). (TIF) [file pone.0075358.s006.tif]

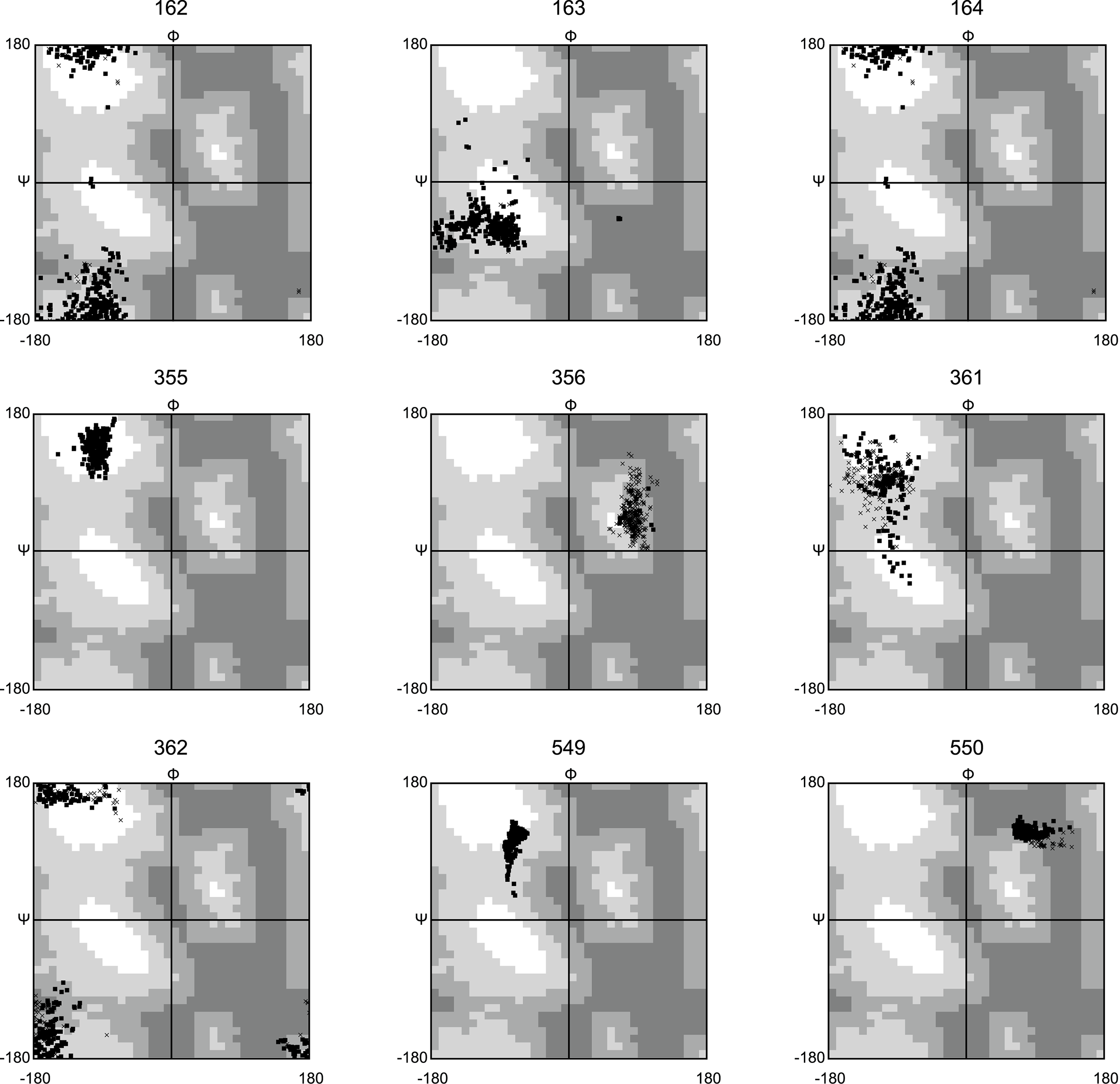

Supplement: Figure S7 — Ramachandran plot of top pNP-GAL mutants. 50 of the top mutants from each of the pNP-GAL libraries were examined. “Core” (white), “allowed” (off white), “generous” (gray), and “outside” (dark gray) regions of the Ramachandran plot were determined by Morris et al. (1992). Results show that glycine residues (crosses) are frequently observed in the “generous” or “outside” regions of the map. Alternatively, the other 19 standard amino acids (squares) are much less frequently observed in the “generous” or “outside” regions. Glycine residues can avoid some of the steric repulsion that is more difficult to avoid for residues with a Cβ. While other amino acids can undergo contortions in their side chain to avoid a strong steric clash, mutation to a glycine residue is more favorable. (TIF) [file pone.0075358.s007.tif]
