# Supplementary material for: OptZyme: Computational Enzyme Redesign Using Transition State Analogues
Source: PLoS One. 2013 Oct 7;8(10):e75358. doi: 10.1371/journal.pone.0075358 (PMC3792102; doi:10.1371/journal.pone.0075358)
Supplement: Table S1 — Gas phase energies from QM cluster model of GUS active site. The gas phase energies are reported for the cluster model of the active site with the backbone of all residues constrained, as well as the ASN 466 sidechain. The calculated energies are relative to the calculated “Intermediate 2 (E)” energy. Each of the three structures corresponds to structures identified in Figures 4 and 6. This correspondence is indicated by each structure’s one-letter label. (DOC) [file pone.0075358.s008.doc]

| **Structure** | **Gas phase energy (kcal/mol)** |
| --- | --- |
| Bound Substrate (A) | 27.9 |
| Intermediate 1 (C) | 23.1 |
| Intermediate 2 (E) | 0.0 |
