# Supplementary material for: OptZyme: Computational Enzyme Redesign Using Transition State Analogues
Source: PLoS One. 2013 Oct 7;8(10):e75358. doi: 10.1371/journal.pone.0075358 (PMC3792102; doi:10.1371/journal.pone.0075358)
Supplement: Table S2 — Primers Used for Switching GUS Specificity. (DOC) [file pone.0075358.s009.doc]

| **Primers to amplify WT-gusA** | |  |
| --- | --- | --- |
| Primer 1.1 | His-gusA-sense-primer-BamHI (5'-3') | CGGGATCCTTAAGAAGGAGATATACCATGGGCAGCAGCCATC |
|  |  | BamHI RBS |
| Primer 1.2 | His-gusA-antisense-primer-EcoRI (5'-3') | GCGAATTCTGCAGTCATTGTTTGCCTCCCTGCT |
|  |  | EcoRI |
| Primers to delete BamHI in gusA | |  |
| Primer 2.1 | For_deltaBamHI | ATCCATCGCAGCGTAATGCTCTACACCA |
| Primer 2.2 | Rev_deltaBamHI | AGCATTACGCTGCGATGGATTCCGGCATAGTTAAAGAAATC |
|  |  |  |
| Primers to convert GUS R2.8 to R3 by oePCR | |  |
| Primer 3.1 | Rev-GUS-oePCR-K568Q | GTCGCGAGTGAAGATCCCTTGCTTGCTACCGCCAACGCGCA |
| Primer 3.2 | Fwd-GUS-oePCR-K568Q | AAGGGATCTTCACTCGCGACCGCAAAC |
