# Supplementary material for: OptZyme: Computational Enzyme Redesign Using Transition State Analogues
Source: PLoS One. 2013 Oct 7;8(10):e75358. doi: 10.1371/journal.pone.0075358 (PMC3792102; doi:10.1371/journal.pone.0075358)
Supplement: Text S1 — Detailed Discussion of QM Calculations. (DOC) [file pone.0075358.s010.doc]

A cluster model of the GUS active site was constructed, including residues D163, E413, N466, R467, and E504 along with the substrate. Energies were obtained for minimized structures *A*, *C*, and *E* (italicized letters correspond to labels from Figures 4 and 6) using Jaguar at the B3LYP/6-31G+** level (Table S1). Quadratic synchronous transit TS searches between *A* and *C* were unsuccessful. Intermediate *C* contains a carbenium ion, which suggests a carbenium ion-like TS between *A* and *C*. Similar reactions have been shown to have relatively flat potential energy surfaces about the carbenium ion leading to difficulty in the characterization of the TS , which is consistent with this result. Quadratic synchronous transit TS searches between structures *C* and *E* found only lower energy structures. As sampling of this portion of the potential energy was sufficiently dense, we conclude that this reaction step is nearly barrier-less. Confirmation of minima or maxima via vibrational modes was difficult due to the constraints necessary to mimic active site behavior, which introduced nonphysical vibrational modes.

1. Janik MJ, Davis RJ, Neurock M (2006) A quantum chemical study of tertiary carbenium ions in acid catalyzed hydrocarbon conversions over phosphotungstic acid. Catalysis Today 116: 90-98.
