# Supplementary material for: OptZyme: Computational Enzyme Redesign Using Transition State Analogues
Source: PLoS One. 2013 Oct 7;8(10):e75358. doi: 10.1371/journal.pone.0075358 (PMC3792102; doi:10.1371/journal.pone.0075358)
Supplement: Text S2 — Derivation of Equations 4 & 5 . (DOC) [file pone.0075358.s011.doc]

Equation 3 can be re-written by treating the fast reaction steps (substrate binding and conversion of intermediate I_1_ to I_2_ and I_2_ to products) as equilibrated (Equation S1). The reaction sequence and reaction rate can then be written using the transformation of the bound substrate to intermediate I1 as the rate-limiting step (Equation S2).

(S1)

(S2)

Equilibrium constants, *K_A_* and *K_P_*, are defined using Equations S3 and S4, respectively. Equation S2 can also be re-written in terms of *K_A_*, *K_P_*, as well as unbound enzyme, product, and substrate concentrations (Equation S5).

(S3)

(S4)

(S5)

Using a site balance on the enzyme, Equation S6, the concentration of unbound enzyme, *[E]*, can be expressed (Equation S7) through the substitution of the expressions for *K_A_* and *K_P_* from Equations S3 and S4, respectively.

(S6)

(S7)

The site balance, Equation S6, presumes the concentrations of I_2_ and bound product are small. Substituting the expression for *[E]* from Equation S7 into Equation S5 allows for the expression of the rate in terms of measurable quantities (Equation S8).

(S8)

This expression can be further simplified (Equation S9) by assuming the equilibrium of product release lies far to the right (large *K_P_* and/or small *[P]*). In other words, *K_P_* is large.

(S9)

The definition of equilibrium dictates that the forward reaction rate must equal the reverse reaction rate (Equation S10).

(S10)

Equations S12 (Equation 4) and S13 (Equation 5) can be expressed by comparison of Equation S9 with the standard Michaelis-Menten equation (Equation S11). Equation S12 is simplified to show how K_M_ can be related to the reciprocal of *K_A_* via comparison of equations S9 and S11 and expressed in terms of the ratio of rate constants that compose *K_A_* (Equation S10).

(S11)

(S12)

(S13)
