# Supplementary material for: OptZyme: Computational Enzyme Redesign Using Transition State Analogues
Source: PLoS One. 2013 Oct 7;8(10):e75358. doi: 10.1371/journal.pone.0075358 (PMC3792102; doi:10.1371/journal.pone.0075358)
Supplement: Text S3 — Experimental Methods for KM Estimation from Cell Lysates. (DOC) [file pone.0075358.s012.doc]

***Plasmid construction***

WT *gusA* and previously described *gusA* mutants gusA-R2 and gusA-R2.8 cloned into vector pET28 were obtained from Matsumura . In these constructs, all *gusA* genes include N-terminal fusion to a 6×His-tag. The *gusA* genes were amplified by PCR using Primer 1.1 and Primer 1.2 (refer to Table S2 for a list of all PCR primers used). A BamHI restriction site within the *gusA* ORF was removed by silent mutation using overlap extension PCR (oePCR) with gene fragments amplified with Primers 1.1, 1.2, 2.1 and 2.2, following a standard protocol . The amplified *gusA* genes were then digested with BamHI and EcoRI, gel-purified, and ligated into plasmid pCWori, also digested with BamHI and EcoRI. The ligation products were transformed into *E. coli* MC1061 competent cells through electroporation. Cells were grown in SOC medium for 45 minutes and then spread on the LB plate containing 100 mg/mL ampicillin.

Single colonies representing unique transformed clones were picked and used to inoculate fresh LB medium with 100 μg/mL ampicillin. The overnight cultures were harvested to extract plasmids using a Qiaprep@Spin Miniprep kit (Qiagen, CA) for sequencing and restriction digestion by BamHI and/or EcoRI, confirming proper construction of each plasmid. The resulting plasmids are pCWori-gusA, pCWori-gusA-R2 and pCWori-gusA-R2.8. pCWori-gusA-R3 was constructed from pCWori-gusA-R2.8 through oePCR with Primers 1.1, 1.2, 3.1 and 3.2, adding one amino acid (AA) substitution as described . These plasmids encode:

pCWori-gusA: WT *E. coli* *β*-glucuronidase, N-terminal 6xHis-tag

pCWori-gusA-R2: AA substitutions T509A, D531E, S557P, N566S

pCWori-gusA-R2.8: AA substitutions T509A,  S557P,  N566S

pCWori-gusA-R3: AA substitutions T509A,  S557P,  N566S,  K568Q

***Protein expression and cell lysis***

Plasmids were transformed into *E. coli* JW1609. Colonies were picked from freshly transformed clones and inoculated in 1-mL LB and grown overnight to saturation. These cultures were then diluted 100-fold into new LB cultures. The new 1-mL cultures were grown for 2 hours before being induced with 100 µM IPTG for 4 hours. The induced cultures were pelleted by centrifugation at 3000 rpm for 10 minutes. The supernatants were discarded and the pellets were resuspended in 100 µL of 50 mM Tris-HCl with 1 mg/mL lysozyme and 2 U/mL DNase I. After 15 minutes of incubation at room temperature, the cell lysates were centrifuged at 2200xg for 20 minutes and 60 µL of each supernatant was transferred into 96-well assay plates.

***Enzyme activity assays***

Hydrolysis of pNP-GAL and pNP-GLU was monitored as follows: 40 µL of appropriate concentrations of stock solutions of pNP-GAL/pNP-GLU was added to 60 µL of lysate supernatant in 96-well plates (the final substrate concentrations were varied in different wells, until Vmax or the solubility limit was reached). The absorbance at 405 nm (corresponding to free pNP) was measured every minute for 10 minutes. Background or non-enzymatic absorbance changes were determined from control culture lysates in which the *gusA* gene was absent from the expression vector. Background activity was negligible but nonetheless subtracted from all data used for kinetic parameter determination. The initial, linear rate was used for calculating the initial activity of each variant at each concentration of pNP-GAL/pNP-GLU. The resulting data for each variant was used to determine the K_M_ for pNP-GAL/pNP-GLU by fitting to the Michaelis-Menten equation by nonlinear regression analysis.

Representative results are presented as Figures S5 and S6. Kinetic parameters represent the average from at least two independent sets of measurements.

1. Matsumura I, Ellington AD (2001) In vitro evolution of beta-glucuronidase into a beta-galactosidase proceeds through non-specific intermediates. J Mol Biol 305: 331-339.

2. Heckman K, Pease L (2007) Gene splicing and mutagenesis by PCR-driven overlap extension. Nature Protocols 2: 924-932.
