# Supplementary material for: OptZyme: Computational Enzyme Redesign Using Transition State Analogues
Source: PLoS One. 2013 Oct 7;8(10):e75358. doi: 10.1371/journal.pone.0075358 (PMC3792102; doi:10.1371/journal.pone.0075358)
Supplement: Text S4 — Prevalence of Small Amino Acids in OptZyme Results. (DOC) [file pone.0075358.s013.doc]

Figures 12 and 13 indicate that there is a strong tendency for OptZyme to mutate to glycine across each of the six libraries despite the additional constraints imposed. This tendency can be partially explained by the backbone flexibility of smaller amino acids. Figure S7 shows the Ramachandran plot for the top 50 mutants in each pNP-GAL library (200 total mutants; pNP-GLU results were similar, unpublished data), with the statistically preferred regions for residues that include a C_β_ indicated . Figure S7 shows that position 356 lies within the disallowed region of the map so glycine must be selected to avoid a steric clash. Similarly, mutation to glycine, alanine, or serine can alleviate the steric clash caused by the backbone conformation at position 550. Due to the highly-packed active site of GUS, mutations leading to larger side chains should be complemented with other size reducing mutations to avoid a steric clash. These two effects may explain the majority of the glycine and alanine (and perhaps to a lesser extent, serine) mutants in the results.

1. Morris AL, MacArthur MW, Hutchinson EG, Thornton JM (1992) Stereochemical quality of protein structure coordinates. Proteins-Structure Function and Bioinformatics 12: 345-364.
